# Prognostic significance of calcium signaling-related genes in bladder cancer and the role of ATP2B4 in regulating mitochondrial calcium ion levels via the VDAC1/MCU pathway

**DOI:** 10.3389/fimmu.2026.1561666

**Published:** 2026-02-06

**Authors:** Lulu Zhang, Yu Gong, Jiajun Chen, Mengyao Li, Xiurong Wang, Weihao Wang, Qiannan Ding, Yulei Li

**Affiliations:** 1 Medical Research Center, Shaoxing People’s Hospital, Shaoxing, Zhejiang, China; 2 Department of Urology, Nanchang People’s Hospital, Nanchang, China; 3 Department of Urology, Shaoxing People’s Hospital, Shaoxing, Zhejiang, China; 4 Department of Pathology, Shaoxing People’s Hospital, Shaoxing, Zhejiang, China; 5 School of Basic Medical Sciences, Fujian Medical University, Fuzhou, Fujian, China; 6 School of Medicine, Shaoxing University, Shaoxing, Zhejiang, China

**Keywords:** ATP2B4, biomarker, BLCA, calcium signaling, immunotherapy, VDAC1/MCU

## Abstract

**Background:**

Calcium signaling, as a ubiquitous intracellular signal in eukaryotes, has been impacted in multiple biological processes encompassing tumorigenesis. Nevertheless, the integrated investigations on the function and prognostic value of genes correlated to calcium signaling in bladder cancer (BLCA) were still lacking.

**Methods:**

The transcriptome data and clinical data from BLCA patients were obtained from TCGA and GEO databases. Genes associated with calcium signaling that are differentially expressed in normal and malignant tissues were identified. Cox analysis and the least absolute shrinkage and selection operator (LASSO) analysis were employed to identify prognostic genes and develop a prognostic signature. The tuning parameter (λ) for LASSO regression was determined by cross-validation. The outcomes were then confirmed using an external independent dataset (GSE32894). The prognostic signature’s reliability was assessed utilizing Kaplan-Meier, PCA, t-SNE, and ROC analyses. Furthermore, both univariate and multivariate Cox regression studies were undertaken to ensure if the prognostic signature functioned as an autonomous prognostic indication. Moreover, we examined the connection between the immune cell infiltration, the tumor mutation burden (TMB), and the prognostic signature. The Genomics of Drug Sensitivity in Cancer (GDSC) database and the IMvigor210 dataset were deployed to forecast the treatment reactions of the prognostic signature. Ultimately, the functionality of ATP2B4 was confirmed by *in vitro* and *in vivo* tests.

**Results:**

Thirty-two differentially expressed calcium signaling-correlated genes were identified in the TCGA dataset. A prognostic signature containing six genes (ATP2B4, BDKRB2, EDNRA, PDGFRA, EGFR, and ADCY7) was ascertained to anticipate the overall survival of BLCA. Furthermore, a nomogram containing risk scores with age was developed to anticipate the BLCA patient’s prognosis. In addition, patients among the high- and low-risk groups displayed significant variation in TMB, immune infiltration landscape, and response to chemotherapy and immunotherapy. ATP2B4 has been recognized as a pivotal oncogenic gene. The suppression of ATP2B4 results in elevated cytoplasmic calcium ion(Ca^2+^) concentrations, which in turn activate the VDAC1/MCU pathway. This activation facilitates the transfer of Ca^2+^ from the cytoplasm to the mitochondria, culminating in mitochondrial Ca^2+^ overload and ultimately inducing apoptosis in bladder urothelial carcinoma (BLCA) cells.

**Conclusions:**

Collectively, we have developed a unique genetic signature that is based on genes associated with calcium signaling. This signature possesses the capacity to precisely anticipate the survival prognosis and therapeutic response of BLCA patients and might have a crucial role in guiding clinical treatment. Furthermore, ATP2B4 has been identified as a crucial oncogenic gene. The downregulation of ATP2B4 leads to mitochondrial Ca^2+^ overload, ultimately resulting in apoptosis of BLCA cells.

## 1 Introduction

Bladder cancer (BLCA) is one of the pervasive urologic carcinomas, with an incidence and mortality second only to the prostate. In 2020, BLCA will be responsible for more than 500,000 newly diagnosed cases and 200,000 fatalities (1). Although encouraging advancements have been made in treatment, including the application of laparoscopic and robotic surgery, immune checkpoint inhibitor (ICI) therapy, and other therapeutic methods, the overall survival rate of BLCA patients is still below expectations. Hence, further research is essential for identifying novel biomarkers associated with early diagnosis and prognosis.

Calcium ion (Ca^2+^), the highest concentration of ions in mammals, was mainly stored in bones in the form of CaPO3 (hydroxyapatite) (2). Ca^2+^, a ubiquitous second messenger, is in essential for numerous physiological and pathological processes such as cell growth, differentiation, gene transcription, inflammation, proliferation, focal adhesion, autophagy, and apoptosis (3–7). The application of Ca^2+^ in cancer treatment was first reported by Das (8). Subsequently, accumulating study has confirmed that Ca^2+^ is essential in developing and progressing the cancer. Huang et al. have observed that the serum calcium level in BLCA patients might function as a useful indication for anticipating bone metastasis (9). ITPR3, a member of the most extensive Ca^2+^ channels, was over-expressed in various types of cancer, including BLCA, and ITPR3 knockdown might decline the proliferation, invasion, lung metastasis, and cancer stemness of BLCA cells by NF-ĸB/CD44 signaling pathway (10). The ZNF503-AS1/GATA6/SLC8A1 signaling pathway could alleviate the malignant transformation of BLCA cells through elevating intracellular Ca^2+^ concentrations (11). Bee venom (BV) accelerated the apoptosis of BLCA cells by a Ca^2+^-modulated intrinsic death pathway (12). However, until now, there was no relevant integrated research on the involvement and predictive significance of the genes correlated with Ca^2+^ signaling in BLCA.

Intracellular Ca²^+^ homeostasis is frequently disrupted in cancer cells, and such alterations can significantly contribute to tumor initiation, progression, angiogenesis, and metastasis (13, 14). Plasma membrane calcium ATPases (PMCA/ATP2B) meticulously regulate intracellular Ca²^+^ concentration by actively pumping Ca²^+^ out of the cytoplasm into the extracellular space, thereby preserving the remarkably low cytosolic Ca²^+^ concentration. Numerous studies have documented alterations in PMCA expression across a wide array of cancer types (14). Voltage-dependent anion channels (VDACs), primarily located in the mitochondrial outer membrane (MOM), play a crucial role in regulating mitochondrial homeostasis and orchestrating various cell death pathways, such as apoptosis, autophagy, and ferroptosis (15, 16). VDAC1, the most abundant of the three VDAC isoforms, is widely recognized for its key role in regulating cell death through interactions with various proteins, including anti-apoptotic proteins such as Bcl-2, Bcl-xL, and hexokinase (HK1 and HK2) (17), proapoptotic proteins such as BH3-interacting domain death agonist (BID) and Bcl-2-associated X protein (Bax) (18, 19). The mitochondrial calcium uniporter (MCU) complex, a substantial assembly, consists of pore-forming subunits and regulatory components like MCU, MICU1, MICU2, MCUb, and EMRE, with MCU facilitating enhanced calcium transport (20). For mitochondrial calcium uptake to occur, Ca^2+^ must traverse the mitochondrial membrane to ultimately reach the mitochondrial matrix (21). Calcium uptake in the mitochondrial matrix is regulated by highly selective MCU channels.

In this investigation, we comprehensively ascertained the calcium-linked genomic expression pattern in BLCA specimens from The Cancer Genome Atlas (TCGA) database and found thirty-two differentially expressed calcium-linked genes (DE-CaRGs), including nine calcium-linked genes (CaRGs) associated with prognosis. A risk signature comprising six CaRGs (ATP2B4, BDKRB2, EDNRA, PDGFRA, EGFR, and ADCY7) was established and validated, which exhibited a favorable predictive ability for outcomes, response to chemotherapy and immunotherapy of patients with BLCA. Moreover, the prognostic signature exhibited a substantial correlation with unfavorable clinical parameters and might function as an autonomous hazardous factor for BLCA patients. Moreover, the signature exhibited associations with cancer signaling pathways, tumor mutation, and immune cell infiltration. Subsequently, further *in vitro* and *in vivo* studies were conducted to validate the impact of ATP2B4 on BLCA cells. The down-regulation of ATP2B4 expression can activate the VADC1/MCU signaling pathway, resulting in mitochondrial Ca^2+^ overload and subsequently inducing cell apoptosis.

## 2 Materials and methods

### 2.1 Data acquisition

Whole-transcriptome profiling data of BLCA patients were downloaded from TCGA (https://portal.gdc.cancer.gov/), along with the corresponding clinical information (age, gender, tumor grade, tumor, T, and N stages in addition to survival time and status). Typically, 178 calcium-related genes (CaRGs) were obtained from gene set “KEGG_CALCIUM_SIGNALING_PATHWAY” in the Molecular Signature Database (MSigDB; https://www.gsea-msigdb.org/gsea/msigdb). Moreover, GSE32894 was obtained from Gene Expression Omnibus (GEO; https://www.ncbi.nlm.nih.gov/geo/).

### 2.2 Differential expression analysis

The differential expression analysis was executed employing the edger module in the R program. Differentially expressed calcium-related genes (DECaRGs) were found employing the specified criteria: a log2 fold change (FC) > 1 in absolute value between BLCA and normal specimens, and a false discovery rate (FDR)< 0.05. The DECaRGs were shown utilizing a heatmap and volcano plots. Furthermore, the DECaRGs were uploaded to the STRING database (version 12.0, https://string-db.org/), which was used to get protein-protein interaction (PPI) information among the DECaRGs. An integrated score > 0.4 was deemed significant. The visualization of PPI was achieved via Cytoscape software (version 3.8.2). We also conducted a gene ontology (GO) analysis and a Kyoto Encyclopedia of Genes and Genomes (KEGG) research to find the possible roles of DECaRGs. This study was done employing the clusterprofiler tools in R software.

### 2.3 Determination of candidate small molecule drugs

In order to find possible small-molecule medications for treating BLCA patients, the researchers employed the Connectivity Map database (CMAP, https://clue.io/) to pick the candidate pharmaceuticals. The enrichment score was employed to assess the drug impact, with a negative value indicating the potential anticancer activity of the treatment.

### 2.4 Development and verification of a prognostic signature associated with calcium

To determine the differentially expressed calcium‐related genes (DECaRGs) that are linked to prognosis, a univariate Cox regression analysis was conducted to investigate the association between DECaRGs and the BLCA patient’s prognosis in the TCGA dataset. For further analysis, all DECaRGs with a P-value< 0.05 were chosen as candidate genes. Then, we performed at the least absolute shrinkage and selection operator (LASSO) analysis to prevent overfitting and choose the best prognostic DECaRGs for creating the prognostic signature. The risk score (RS) for BLCA patients was computed utilizing the subsequent formula: risk scores= $\sum_{i}^{n}{\text{X}}_{i}\times {\text{Y}}_{\text{i}}$ (where mRNA expression of a gene is denoted by Y and the LASSO analysis coefficient associated with the corresponding gene is signified by X). Patients were classified into low-risk group (LRG) and high-risk group (HRG) based on the RS that corresponded to the midpoint of the risk distribution. Survival studies were performed to assess the variation in prognosis across various groups using the survival and survminer packages in R. Using the survivalROC package, a receiver operating characteristic (ROC) curve was also employed to estimate the predicted signature accuracy. Principal component analysis (PCA) and t-distributed stochastic neighbor embedding (t-SNE) were subsequently utilized to ascertain the distribution characteristics of patients between the two groups. Using the identical methodology, GSE32894 was employed as an external validation dataset to confirm the dependability of our developed signature.

### 2.5 Gene set enrichment analysis, tumor mutation burden, and immune infiltration analyses

GSEA was deployed to ascertain the underlying biological processes associated with the calcium-related signature, utilizing a significance threshold of P-value< 0.05 and FDR<0.25. Meanwhile, we also obtained the tumor mutational information from the TCGA dataset with the application of maftools package in R. The TMB was ascertained by analyzing the individual genes that were mutated in the tumor. The mutation status was categorized into high-risk group (HRG) and low-risk group (LRG). The significance of the tumor immune microenvironment was assessed using several techniques, including TIMER, CIBERSORT, CIBERSORT-ABS, XCELL, QUANTISEQ, EPIC, and MCP-counter. These methods were employed to analyze the extent of immune cell infiltration among 22 distinct leukocyte subsets in various groups. In addition, we conducted an investigation into the connection between RS and well-known immunological checkpoints, including PD-1, CD274, CTLA4, LAG3, HAVCR2, and TIGIT. A p-value less than 0.05 was selected as the statistical criterion.

### 2.6 Response to chemotherapy

In order to examine the variation in chemotherapy sensitivity across various groups, we employed the GDSC database to compute the IC50 values, half of the maximum suppressive concentration, for chemotherapeutic drugs. This was done to forecast the sensitivity of chemotherapy treatments employing the pRRophetic package. Statistically significant outcomes were deemed as having P values< 0.05.

### 2.7 Response to immunotherapy

The IMvigor210 dataset, an investigation of atezolizumab in locally advanced or metastatic urothelial carcinoma patients (22), was employed to ascertain the performance of calcium-related signatures for predicting the response to atezolizumab. In addition, GSE19423, an investigation of Bacillus Calmette-Guérin (BCG) in patients with primary pT1 BLCA (23), was performed to ascertain the performance of calcium-correlated signature for anticipating the response to BCG. P values< 0.05 were deemed statistically significant.

### 2.8 Independence of the calcium-correlated signature

Univariate and multivariate Cox regression analyses were performed to ascertain if the calcium-correlated signature functioned as an independent prognostic predictor in BLCA patients, taking into account other standard clinical parameters encompassing age, gender, tumor grade, tumor, T, and N stages. Moreover, we categorized individuals into several subgroups based on their clinical features. We subsequently employed Kaplan-Meier curves to analyze the effectiveness of the calcium-related signature to forecast prognosis within each grouping.

### 2.9 Construction of a nomogram

A nomogram was created employing multivariate analysis to anticipate the 3- and 5-year overall survival on the basis of risk score (RS) and other clinical parameters. Utilizing the calibration curve, the accuracy of survival prediction in the nomogram was ascertained.

### 2.10 Patient sample

Between 2022 and 2024, a total of 20 samples of bladder cancer (BLCA) tissue, along with their corresponding non-cancerous counterparts, were procured from Shaoxing People’s Hospital for immunohistochemical (IHC) staining. Importantly, none of the patients from whom these samples were obtained had received radiation therapy, adjuvant therapy, or preoperative chemotherapy. The collection of these samples was conducted with the informed consent of the patients, and the study received approval from the hospital’s Academic Ethical Committee (ethical approval code: 2022-K-Y-054-01). The research was conducted in accordance with the principles outlined in the Declaration of Helsinki. Detailed clinical data for each patient are provided in **Supplementary Table S1**.

### 2.11 Cell culture, treatments, and siRNA/shRNA transfection

The T24 and 5637 cell lines, originating from human bladder carcinoma (BLCA), were procured from Procell Life Science & Technology Company (Hubei, China). Small interfering RNA (siRNA) was obtained from GenePharma (Shanghai, China), while ATP2B4-underexpressed lentiviruses and short hairpin RNA (shRNA) were sourced from GeneChem (Shanghai, China). Transfections were executed using Lipofectamine 6000 (#C0526, Beyotime), and infections were carried out with the Hitrans AP reagent (GeneChem), adhering to the manufacturer’s protocol. Furthermore, dual siRNAs or shRNAs were specifically designed for each target, as detailed in **Supplementary Table S2**. The composite was subsequently introduced into the cell culture plate. After a 48-hour transfection period, the cells were harvested for further analysis.

### 2.12 Western-blot analysis

Proteins were extracted utilizing RIPA buffers, and their concentrations were quantified using BCA assay kits. The protein solution was resolved via 10% SDS-PAGE and subsequently transferred onto a PVDF membrane. Following the blocking procedure, the PVDF membrane was incubated with primary and secondary antibodies. For the isolation of mitochondria, a mitochondria isolation kit for cultured cells (Medchemexpress, HY-K1060) was utilized.

### 2.13 Antibodies

The following antibodies were used: anti-ATP2B4 (Abcam, ab2783); anti-β-actin (Proteintech, 81115-1-RR); anti-AIF (Proteintech, 17984-1-AP); anti-Cytochrome c Polyclonal (Proteintech,10993-1-AP); anti-Caspase-3 (Proteintech, 40924); anti-Bax (Proteintech, 50599-2-Ig); anti-Bcl-2 (Proteintech, 12789-1-AP); anti-VDAC1 (Abcam, ab14734); anti-MCU (Abcam, ab219827); anti-ki67 (Proteintech, 27309-1-AP).

### 2.14 Cell viability assay

Cell viability was performed by deploying a cell counting kit-8 (CCK-8) assay (CCK-8, MCE, HY-K0301).

### 2.15 Animal experiments

BALB/c mice were obtained from Shanghai SLAC Laboratory Animal Co., Ltd., and maintained under specific pathogen-free conditions at a constant temperature of 23 ± 1°C with a 12-hour light/dark cycle. All animal experiments adhered strictly to the guidelines approved by the Animal Ethics Committee of Shaoxing People’s Hospital. For the BLCA xenograft model, immunodeficient nude mice aged 6 to 8 weeks were subcutaneously injected with T24 cells transfected with either shNC or shATP2B4. The mice were randomly divided into two groups, each comprising six mice. Tumor volume was assessed every five days until day 20. At the conclusion of the experiment, the animals were euthanized via an overdose of thiopental (100 mg/kg). Subsequently, the tumors were excised, weighed, and prepared for histological analysis.

### 2.16 Hematoxylin-eosin staining

The specimens underwent a staining protocol beginning with a 4-minute immersion in hematoxylin staining solution, followed by differentiation with a differentiation solution, and subsequent bluing with a reblue solution. Post-dehydration for a duration of 5 minutes, the specimens were subjected to eosin staining for approximately 5 minutes. The samples were then sealed with neutral gum, and image acquisition was conducted using a panoramic scanner.

### 2.17 Immunohistochemical analysis

For the bright field immunohistochemistry procedure, tissue sections underwent an initial treatment with BIOXALL (Vector Laboratories) for a duration of 10 minutes. This was succeeded by a blocking step and subsequent incubation with primary antibodies. Visualization of secondary staining was achieved using 3,3’-diaminobenzidine (DAB) (Vector Laboratories), and sections were counterstained with hematoxylin (Sigma-Aldrich) before being mounted using Pertex mounting medium. For histological analysis, the tissue sections were deparaffinized and stained with a combination of hematoxylin, eosin, and alcian blue.

### 2.18 Detection of mitochondrial membrane potential

T24 and 5637 cells were seeded into 96-well microplates and subsequently stained with a 1× JC-1 staining solution (Medchemexpress, HY-DY1003) under light-protected conditions for 20 minutes. Following staining, the cells were washed three times with JC-1 staining buffer.

### 2.19 Intracellular reactive oxygen species

Intracellular (ROS) levels were assessed with the fluorescent probe 2’,7’-dichlorodihydrofluorescein diacetate (DCFH-DA), in accordance with a previously reported method (24). In detail, the cells were incubated with 1 mL of DCFH-DA solution per well and maintained in darkness at 37°C for 20 minutes. Subsequently, the cells were washed and observed using an inverted fluorescence microscope (Leica, Wetzlar, Germany). The level of reactive oxygen species (ROS) was quantified by measuring the fluorescence intensity of DCFH. Each experimental condition was assessed in triplicate biological replicates.

### 2.20 Annexin V-FITC apoptosis detection assay

Apoptosis was assessed utilizing the annexin V-FITC assay kit. Following cell processing, the cells were collected and resuspended in flow cytometry tubes. The cell suspension was subsequently stained with a solution comprising 195 μL of binding buffer, 5 μL of annexin V-FITC, and 10 μL of propidium iodide (PI). Flow cytometry (Beckman, Brea, CA, USA) was employed to quantify the degree of apoptosis. The experiment was independently replicated three times, with duplicate samples for each group, resulting in a total of six samples per treatment condition.

### 2.21 Detection of calcium contents

Calcium concentrations within the cytoplasm and mitochondria were assessed utilizing the specific fluorescent probes Fluo-4 AM and Rhod-2 AM, respectively. Following treatment, T24 and 5637 cells were incubated with the appropriate calcium indicators for 20 minutes. The fluorescence intensity, representative of intracellular calcium levels, was recorded using an inverted fluorescence microscope. Three separate fields of view were imaged for each group, and the fluorescence intensity was subsequently quantified.

### 2.22 Statistical analysis

Statistical analyses were performed utilizing R software (version 4.0.5) and GraphPad Prism (version 9.3.1). The Wilcoxon test was employed to evaluate differences between the high-risk group (HRG) and the low-risk group (LRG), with p-values less than 0.05 considered statistically significant. Results are presented as the mean ± standard error of the mean (SEM), based on a minimum of three independent experiments. For datasets with a normal distribution, as confirmed by the Shapiro–Wilk test, between-group comparisons were conducted using the two-tailed t-test. In cases where the data did not exhibit a normal distribution, the Mann–Whitney U test was utilized. All statistical tests were conducted on a minimum of three independent experiments, with statistical significance indicated by p-values: *p < 0.05, **p < 0.01, ***p < 0.001.

## 3 Results

### 3.1 Determination of differentially expressed calcium‐related genes

**Figure 1:**
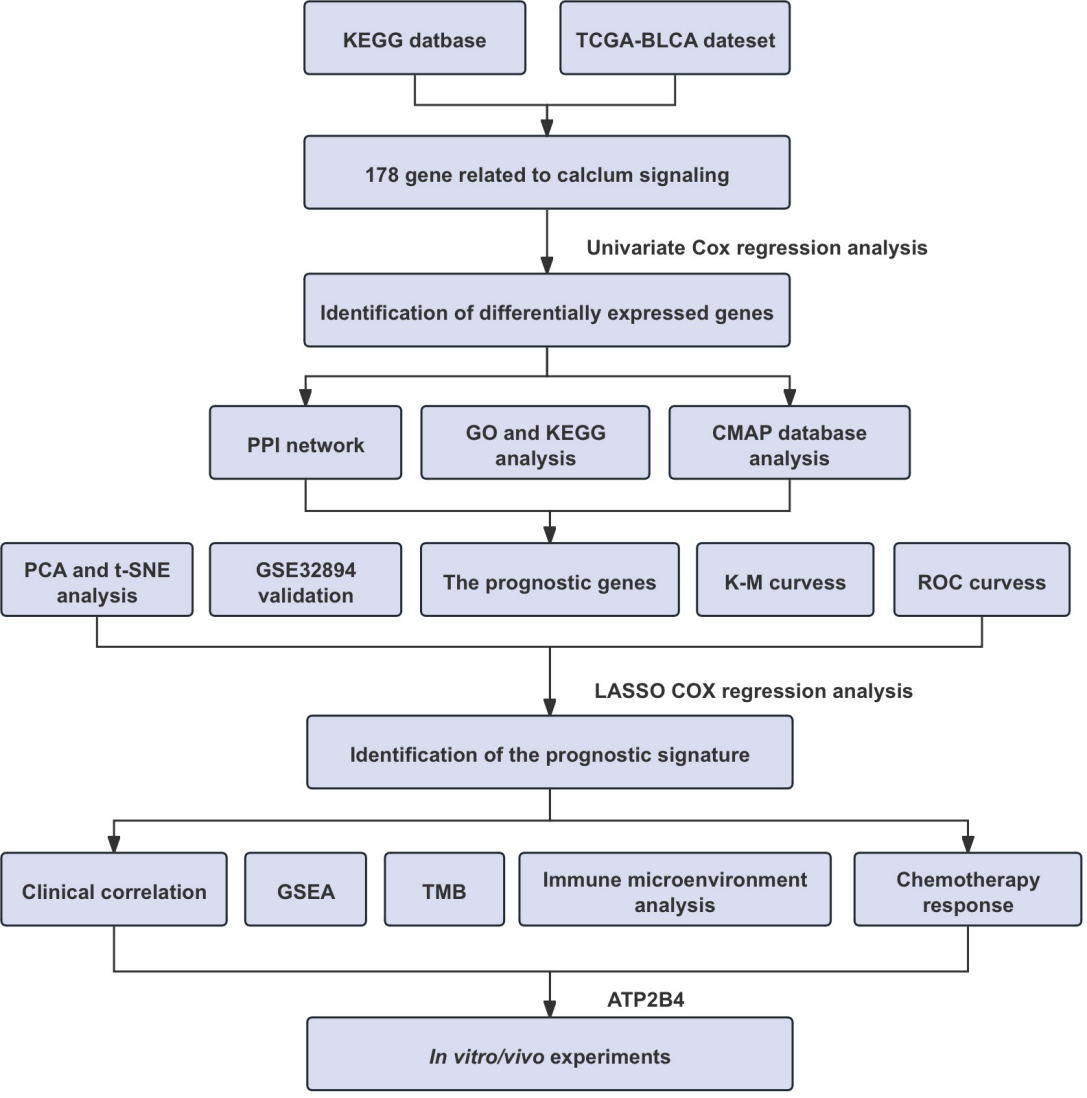
The flow chart of our research.

**Figure 2:**
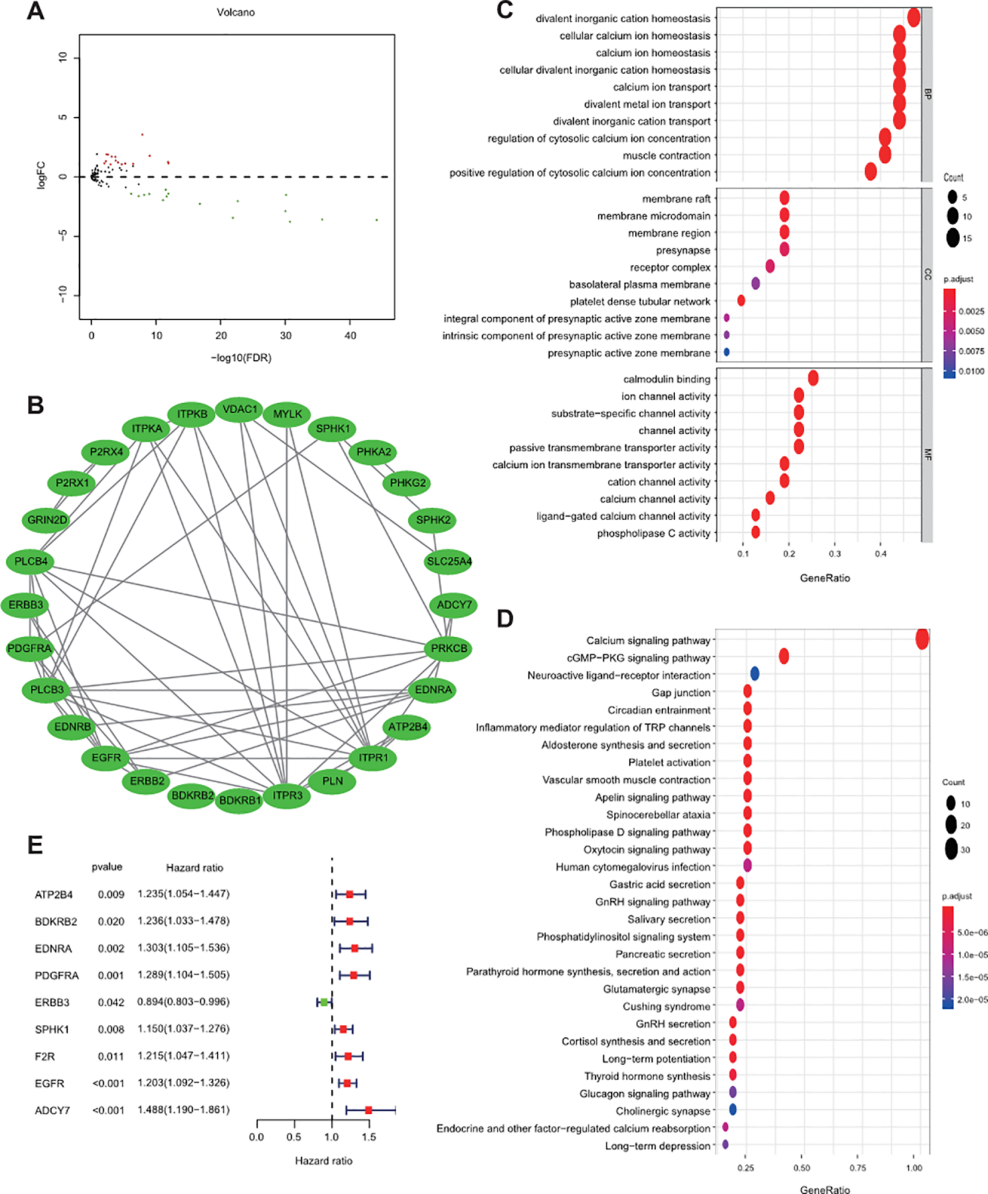
Identification of the differentially expressed calcium‐correlated genes. **(A)** Volcano map manifested differentially expressed calcium‐related genes (DECaRGs); **(B)** Protein-protein interaction network; **(C)** GO analysis; **(D)** KEGG analysis; **(E)** Identification of the prognostic genes via univariate Cox regression analysis.

**Figure 1** depicts the comprehensive methodology used in our investigation. The transcriptome data of 178 calcium-linked genes (CaRGs) were retrieved from the TCGA dataset. These genes were compared between 412 BLCA and 19 normal bladder specimens. A total of 32 DECaRGs were discovered using the criteria of an FDR< 0.05 and an absolute log2 FC > 1. Among these, 16 CaRGs were found to be downregulated, while the other 16 CaRGs were upregulated. The volcano plot was employed to display the DECaRGs expression profile (**Figure 2A**).

### 3.2 Functional enrichment analyses and PPI network

To ascertain the relationship between differentially expressed calcium‐related genes (DECaRGs), a PPI network composed of 28 nodes and 55 edges was created and shown utilizing Cytoscape (**Figure 2B**). Furthermore, to get a deeper knowledge of the possible biological processes and pathways associated with DECaRGs, we conducted GO and KEGG analyses using a set of 32 DECaRGs. The findings of GO analysis revealed that the DECaRGs mostly participated in maintaining the balance of Ca^2+^ inside cells, controlling the concentration of Ca^2+^ in the cytoplasm, transporting Ca^2+^ across membranes, binding to calmodulin, and functioning as calcium channels (**Figure 2C**). Furthermore, the KEGG analysis manifested that the DECaRGs were mostly connected with the calcium pathway, cGMP−PKG signaling pathway, Gap junction, and inflammatory mediator modulation of TRP channels (**Figure 2D**). These data suggest that lipid metabolism may be essential in developing BLCA.

### 3.3 Small molecular drugs

In order to increase the therapeutic impact of BLCA, the CMAP database was used to discover potential medications using the differentially expressed calcium‐related genes (DECaRGs). Ten small molecular compounds with anticancer potential were observed as the best performers (**Table 1**). These medications (tomatine, tyloxapol, Prestwick-1084, mepenzolate bromide, calcium pantothenate, nisoxetine, carbarsone, isoconazole, hydrocotarnine, and harmine) have the ability to slow down the BLCA progression and might be used as new targeted therapies for BLCA therapy.

**Table 1:** The 10 small molecule drugs of CMAP database analyses in bladder cancer.

CMAP name	Mean	Enrichment	*P*	Percent non-null
tomatidine	-0.831	-0.991	0.00026	100
tyloxapol	-0.818	-0.975	0.00129	100
Prestwick-1084	-0.78	-0.971	0.00167	100
hydrocotarnine	-0.823	-0.971	0.00169	100
calcium pantothenate	-0.754	-0.964	0.00288	100
nisoxetine	-0.757	-0.963	0.00312	100
carbarsone	-0.817	-0.962	0.00326	100
isoconazole	-0.783	-0.959	0.00372	100
mepenzolate bromide	-0.733	-0.958	0.00378	100
harmine	-0.787	-0.958	0.0038	100

### 3.4 Creation of low-risk groups signature for anticipating OS

**Figure 3:**
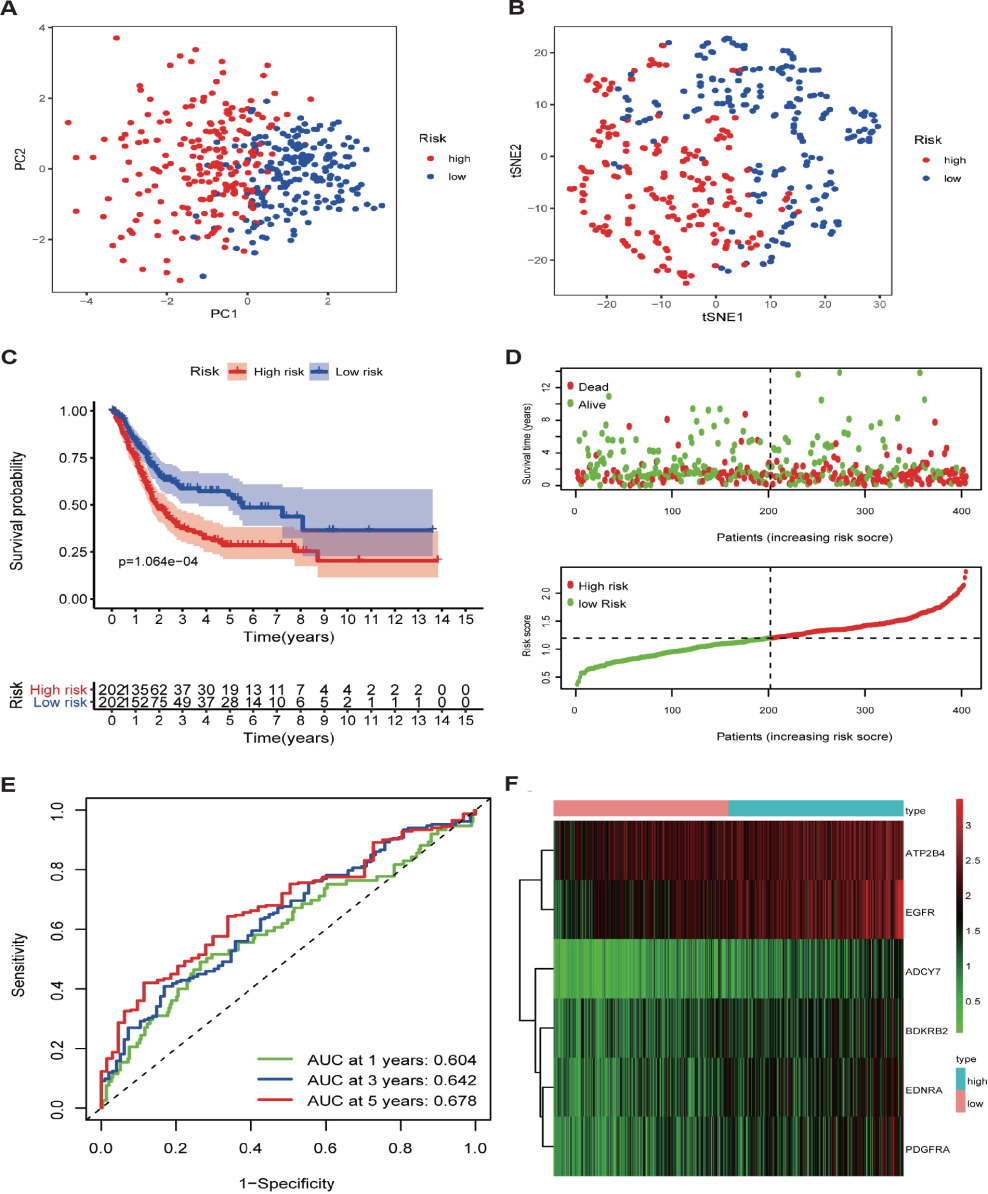
Construction and validation of the prognostic signature in TCGA dataset **(A)** PCA analyses; **(B)** t-SNE analyses; **(C)** Kaplan-Meier survival analysis of BLCA patients between high-risk groups and low-risk groups; **(D)** Distribution of risk score and different patterns of survival status and survival time between the high- and low- risk groups; **(E)** Time-independent receiver operating characteristic (ROC) analysis of the risk score predicting the overall survival; **(F)** Heatmap displayed the distribution of six genes between the high- and low-risk groups.

According to the previous result of differential expression analysis, univariate Cox regression analysis was performed to select differentially expressed calcium‐related genes (DECaRGs) linked to over-survival of BLCA patients in the TCGA dataset and nine DECaRGs were observed (**Figure 2E**). Subsequently, LASSO Cox regression analysis was conducted to additionally estimate prognostic DECaRGs without the overfitting and construct a CaRG-based prognostic signature consisting of ATP2B4, BDKRB2, EDNRA, PDGFRA, EGFR, and ADCY7, and 10-fold cross-validation was used to determine the optimal penalty parameter to prevent overfitting (**Supplementary Figure 1**). The risk score (RS) was generated as follows: RS = (0.084 × ATP2B4 expression) +(0.0278 × BDKRB2 expression) + (0.007 × EDNRA expression) +(0.2008 × PDGFRA expression) + (0.1377 × EGFR expression) +(0.1244 × ADCY7 expression). Following this, the patients were categorized into various groups according to their median risk score (RS). PCA and t-SNE analysis exhibited the different dimensions between the two groups (**Figures 3A, B**). The dot plot displaying each patient’s survival status indicated that the high-risk group (HRG) had significantly worse outcomes compared to the LRG (**Figure 3D**). The HRG exhibited heightened expression levels of ATP2B4, BDKRB2, EDNRA, PDGFRA, EGFR, and ADCY7, as depicted in **Figure 3F**. It was observed that patients assigned to the LRG exhibited more favorable outcomes compared to those in the HRG, as evidenced via the Kaplan-Meier curves (**Figure 3C**). The examination of the time-dependent ROC curve manifested that the AUC values for the calcium-linked genes (CaRG) based signature in the TCGA dataset were 0.604, 0.642, and 0.678 for survival at 1, 3, and 5 years, respectively (**Figure 3E**).

### 3.5 Confirmation of the calcium-linked genes signature in GEO dataset

In order to validate the reliability of the CaRG-based signature, we used GSE32894 as a validation set to confirm our prior findings. The BLCA patients in the GSE32894 dataset were allocated high-risk group (HRG) and low-risk group (LRG), employing the same method as before. Consistent with the outcomes shown above, the survival analysis manifested that the LRG patients had significantly extended survival times compared to those in the HRG (**Supplementary Figure 2C**). The PCA and t-SNE analysis manifested the distinct variations in dimensions between the two groups (**Supplementary Figures 2A, B**). The dot plot displaying each patient’s survival status indicated that the HRG had significantly worse outcomes compared to the LRG (**Supplementary Figure 2D**). The time-dependent ROC curve manifested that the AUC values for the CaRG-based signature in the GSE32894 dataset were 0.640, 0.642, and 0.631 for the 1-, 3-, and 5-year survival periods, respectively (**Supplementary Figure 2E**). The HRG exhibited heightened expression levels of ATP2B4, BDKRB2, EDNRA, PDGFRA, EGFR, and ADCY7, as depicted in **Supplementary Figure 2F**.

### 3.6 The five-gene-based model can predict different clinical characteristics.

**Figure 4:**
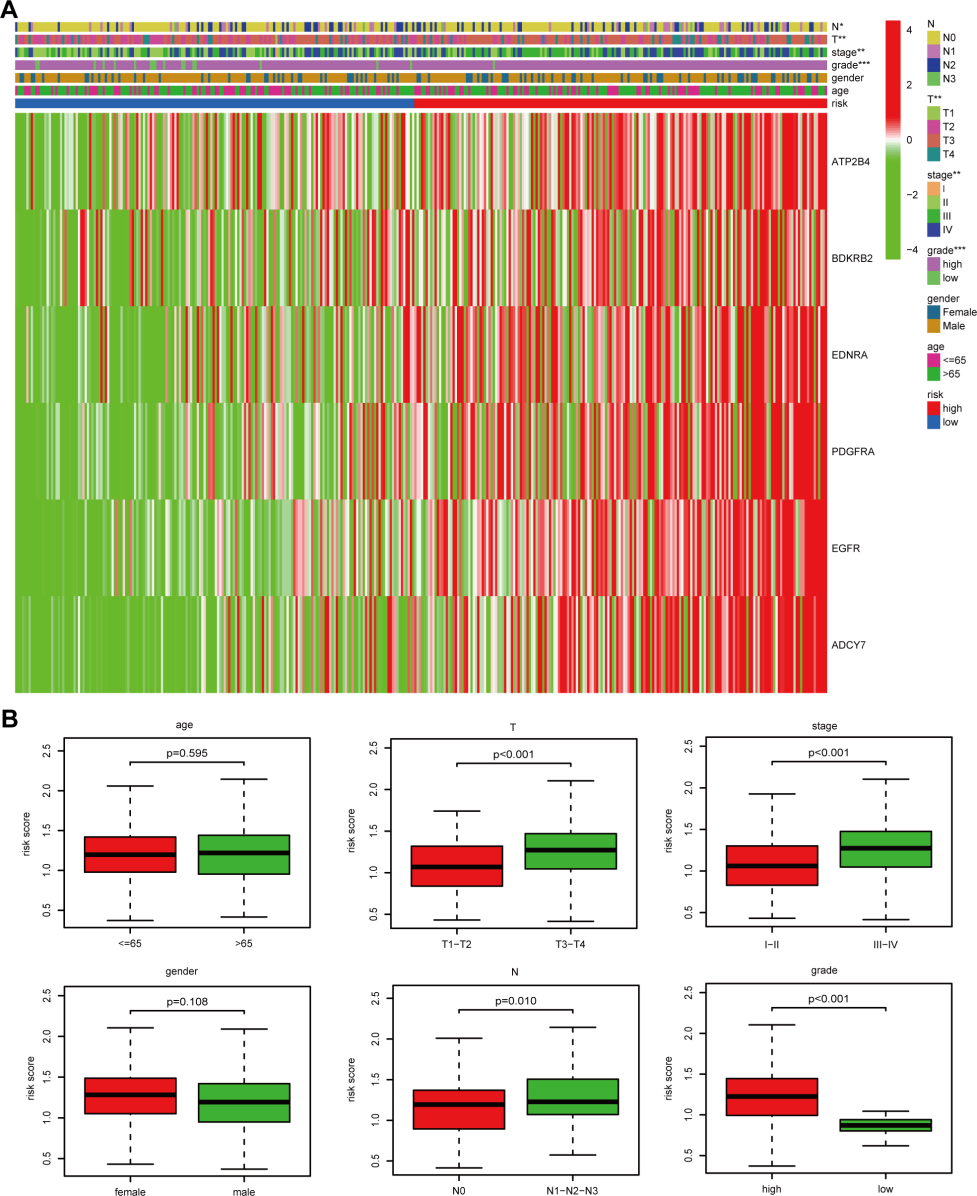
The five-gene-based model can predict different clinical characteristics. **(A)** Correlation analyses between the risk score (RS) and clinicopathological factors shown by heatmap; **(B)** Correlation analyses between the RS and clinicopathological factors shown by boxplot.

To ascertain the connection between risk score (RS) and clinical characteristics encompassing age, gender, tumor grade, tumor, T, and N stages, we conducted an analysis of RS distribution among patients who were classified according to these clinical attributes. Our findings revealed a clear correlation between RS and advanced clinical characteristics, specifically T3-T4, N1-N2-N3, and III-IV stages (**Figures 4A, B**). In addition, to ascertain whether calcium-linked genes (CaRG) based signature could function as an indicator associated with outcomes in subgroups stratified by various clinical features, subgroup analyses were conducted. The results indicated that the survival probability is clearly unfavorable in patients with a high RS, except for those in the T3-T4, N1-N2-N3, and III-IV stages subgroups (**Supplementary Figure 3**).

### 3.7 Construction of a nomogram

**Figure 5:**
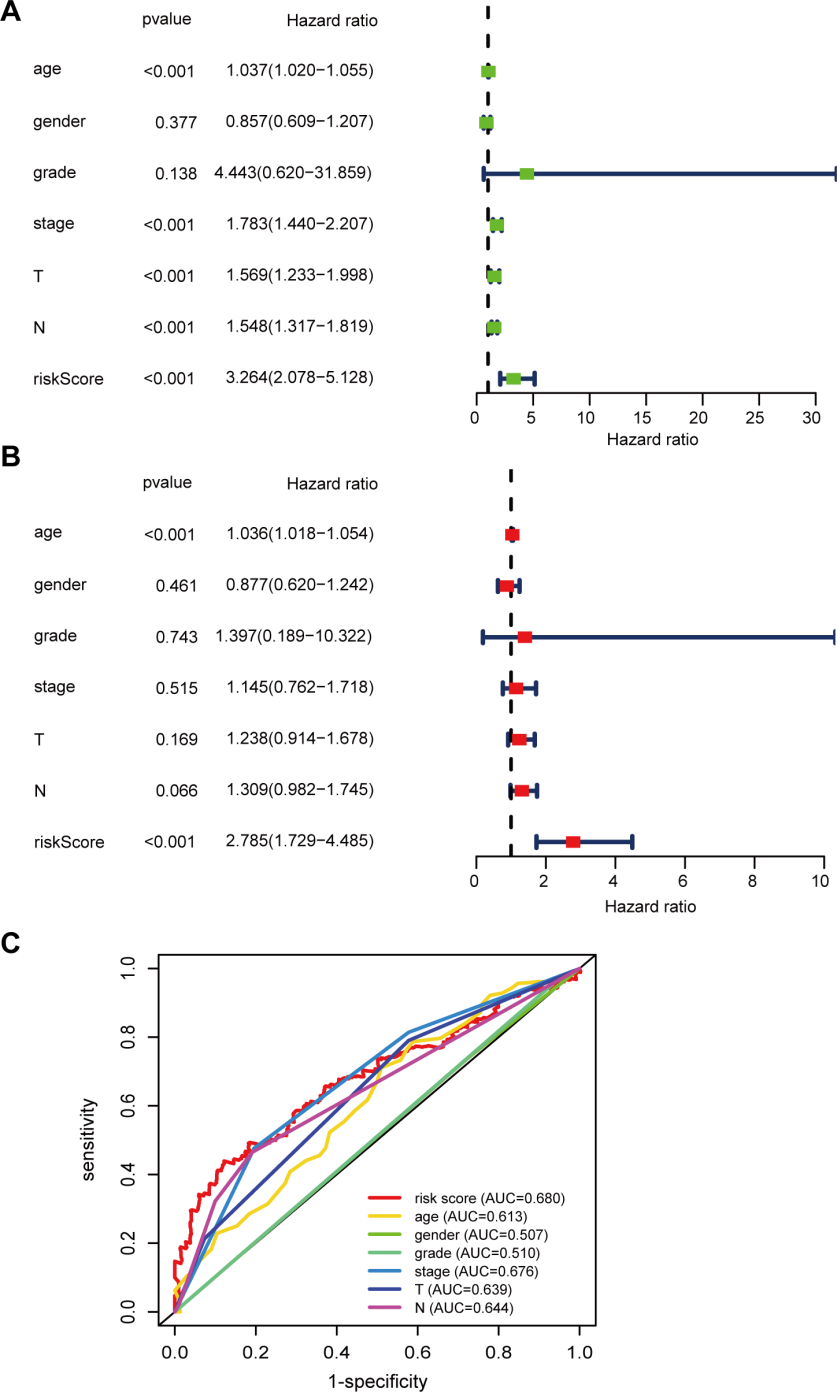
Establishment of a nomogram. **(A)** The correlations between the RS and clinicopathological factors for OS by univariate Cox regression analysis; **(B)** The correlations between the RS and clinicopathological factors for OS by multivariate Cox regression analysis; **(C)** ROC curves of the clinical characteristics and RS.

To ascertain if the six-gene-based signature can be employed as an independent prognostic predictor for BLCA patients, we performed univariate and multivariate Cox regression analysis. The result of univariate analysis indicated the noticeable correlativity between risk score (RS) and prognosis of BLCA patients (HR = 3.264, 95%CI 2.078–5.128, and *P* < 0.001) (**Figure 5A**). Furthermore, the outcome of the multivariate analysis emerged that the RS may function as an independent prognostic indicator (HR = 2.785, 95%CI 1.729–4.485, and *P* < 0.001) (**Figure 5B**). Moreover, the analysis of ROC curves with many parameters also emerged that the AUC value of the RS was 0.68 (**Figure 5C**). This finding suggests that the RS based on calcium-linked genes (CaRG) is more effective than standard clinical prognostic markers in forecasting prognosis. In order to accurately forecast the overall survival of BLCA patients, we developed a nomogram that incorporates both the RS and age variables (**Supplementary Figure 4A**). The calibration curve manifested that the nomogram effectively predicted the prognosis, as shown in **Supplementary Figures 4B, C**.

### 3.8 GSEA

**Figure 6:**
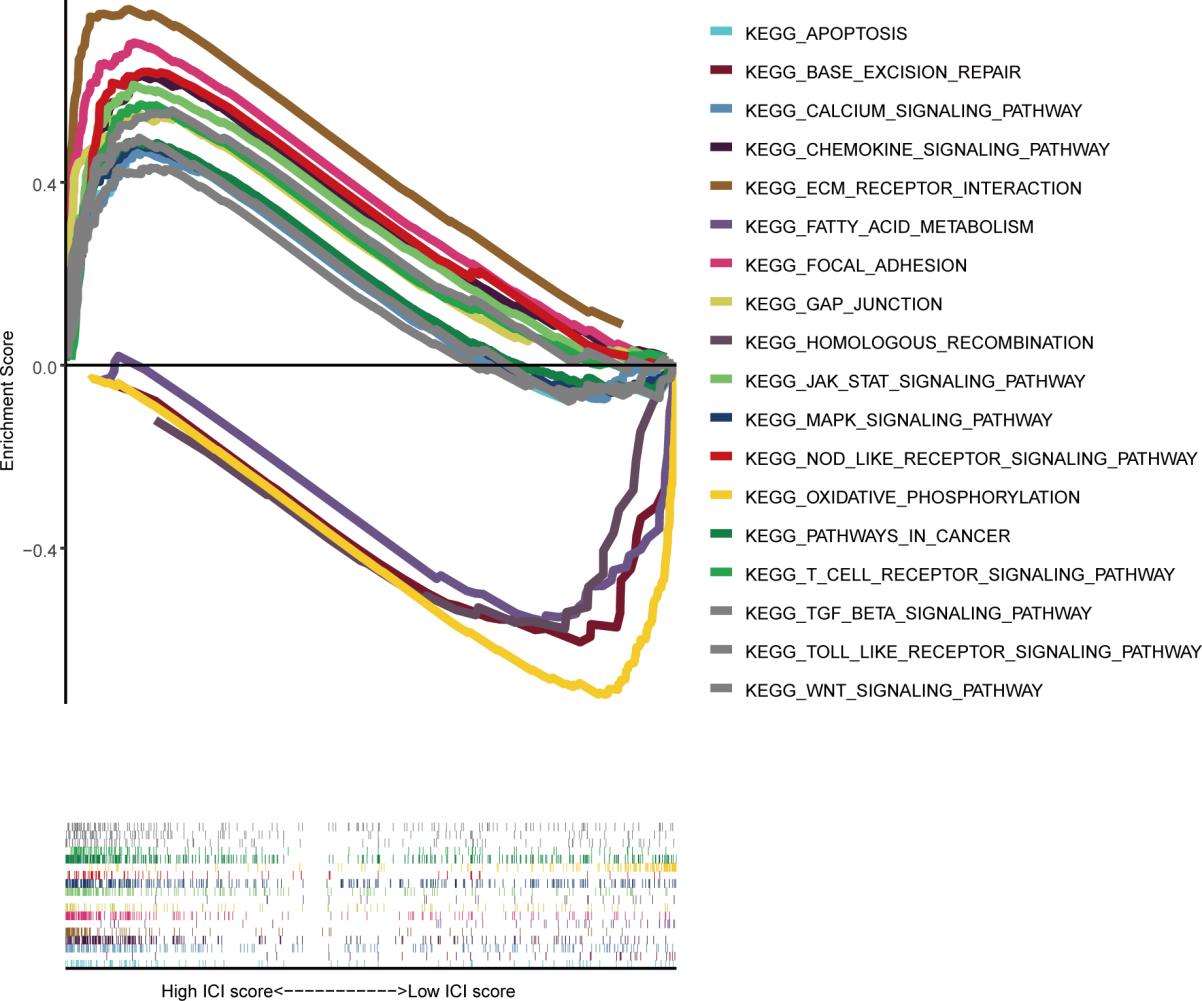
GSEA analysis between the high-risk group (HRG) and low-risk group (LRG); the HRG and LRG.

A GSEA was performed to provide a more detailed explanation of the molecular processes underlying the CaRG-based signature. The GSEA analysis manifested that multiple biological pathways were significantly enriched in the high-risk group (HRG), encompassing focal adhesion, cytokine-cytokine receptor interaction, ECM receptor interaction, GAP Junction, pathway in cancer, NOD-like receptor, chemokine, JAK-STAT, MAPK, Toll-like receptor, T cell receptor, Wnt, calcium, and TGF-β signaling pathways, and apoptosis. Conversely, the low-risk group (LRG) showed high enrichment in oxidative phosphorylation, fatty acid metabolism, base excision repair, and homologous recombination (**Figure 6**).

### 3.9 Tumor mutation burden

**Figure 7:**
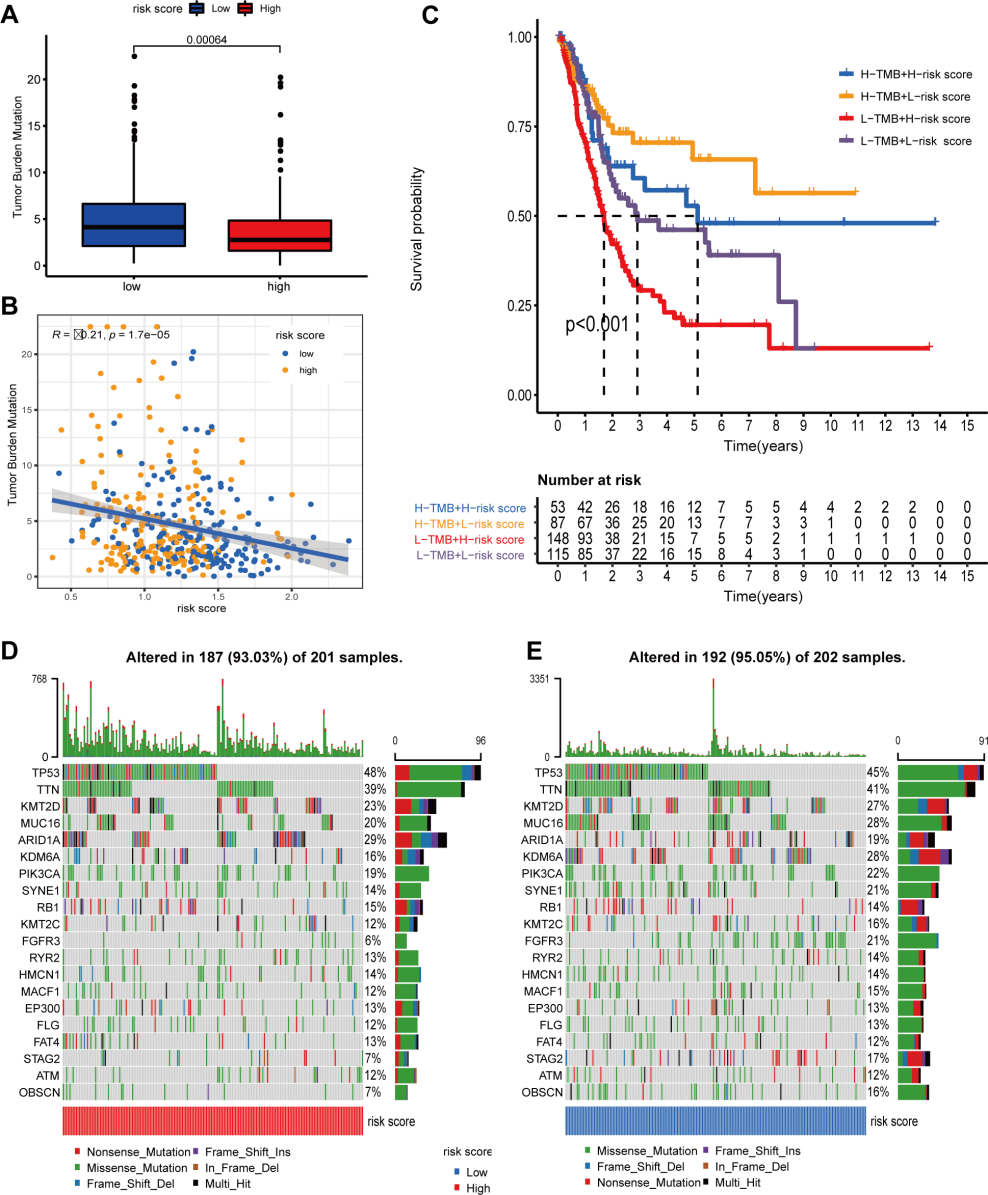
Predictive value of the prognostic signature for immune checkpoint inhibitor (ICI) and Bacillus Calmette-Guérin (BCG) response. **(A)** TMB difference in both risk groups. **(B)** The TMB and risk score (RS); **(C)** Kaplan-Meier curve analysis was shown for OS classified by TMBs **(D, E)** Waterfall plot displayed the genes with high in the HRG **(D)** and LRG **(E)**.

Increasing studies suggested that accumulating somatic mutation variants could enhance the antitumor immune response. Elevated TMB indicated a better response to immune checkpoint inhibitor (ICI) therapy. Therefore, we performed a comprehensive study to investigate the correlation between the risk score (RS) derived by calcium-linked genes (CaRG) and TMB. The outcomes manifested as low-risk group (LRG) had a significantly larger TMB compared to those classified as high-risk. The genes TP53, TTN, KMT2D, MUC16, ARID1A, KDM6A, PIK3CA, SYNE1, RB1, and KMT2C were identified as the top 10 genes with mutations in both the high-risk group (HRG) and LRG (**Figures 7D, E**). Furthermore, the Spearman correlation analysis confirmed a robust negative connection between the RS derived from CaRG and the TMB (**Figures 7A, B**). Furthermore, we discovered that the combined effect of CaRG-based RS and TMB enhanced the ability to forecast the OS of BLCA patients (**Figure 7C**). The findings emerge that the CaRG-based RS has the capacity to function as an indication for predicting the response to immunotherapy.

### 3.10 Immune landscape between two groups

**Figure 8:**
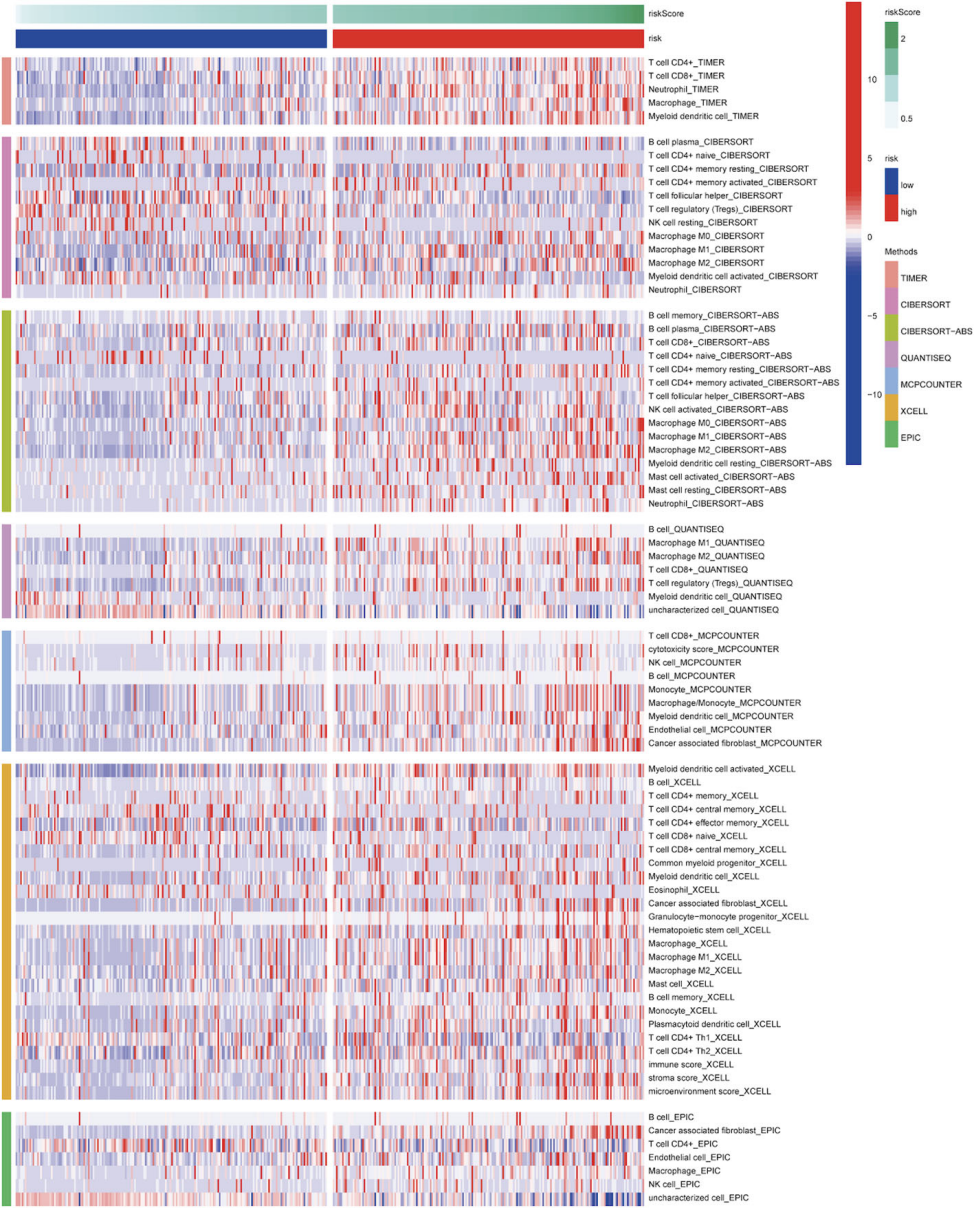
Immune infiltrating cells among high-risk group (HRG) and low-risk group (LRG).

**Figure 9:**
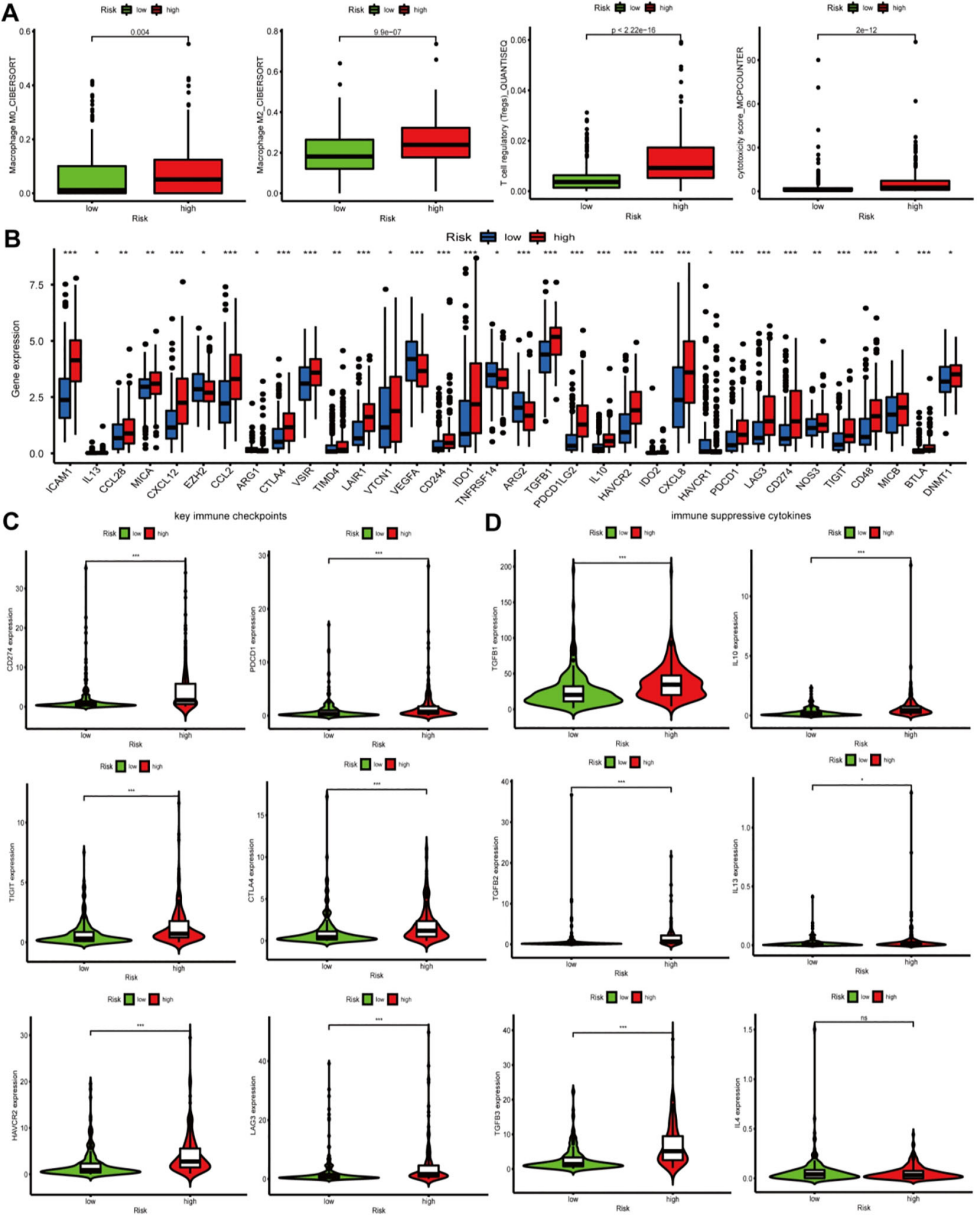
The Connection between the immune microenvironment and the prognostic signature. **(A)** Immune suppressive cells between high-risk group (HRG) and low-risk group (LRG); **(B)** Immune suppressive molecules between HRG and LRG; **(C)** Key immune checkpoints between HRG and LRG; **(D)** Immunosuppressive chemokines between HRG and LRG. *P < 0.05; **P < 0.01; ***P < 0.001.

In order to determine whether the calcium-linked genes (CaRG) based signature might represent the characteristics of the tumor immune microenvironment, we used various algorithms that are related to immune cell infiltration. These algorithms include TIMER, CIBERSORT, XCELL, CIBERSORT-ABS, EPIC, QUANTISEQ, and MCP-counter. The heatmap of immune cell infiltration analyses on the basis of CIBERSORT, MCP-counter, XCELL, TIMER, CIBERSORT-ABS, QUANTISEQ, and EPIC algorithms was emerged in **Figure 8**, which manifested that the risk score (RS) was connected with immune cell infiltration. In addition, immune suppressive cells, including Tregs, Macrophages or M2 Macrophages, neutrophils, and cancer-associated fibroblasts, were mainly enriched in high-risk group (HRG), which suggested that an immunosuppressive phenotype might exist in HRG (**Figure 9A**). Furthermore, our research revealed that immune suppressive molecules were increased in the HRG, indicating that patients in this group had diminished anticancer immune response activities (**Figure 9B**). Subsequently, we conducted a more in-depth analysis of the crucial immunological checkpoint expression (PDCD1, PD-L1, LAG3, CTLA-4, TIGIT, and HAVCR2) and discovered that these immune checkpoints were increased in the HRG (**Figure 9C**). In addition, chemokines linked to the immunosuppressive process (IL-10, IL-13, TGF-β1, -β2, and -β3) have been observed to be elevated in the HRG (**Figure 9D**). The aforementioned data suggests the negative consequences seen in HRG may be caused by the immunosuppressive microenvironment.

### 3.11 Immunotherapy efficacy prediction

To demonstrate the connection between prognostic signature and the effectiveness of immunotherapy in BLCA patients, we divided the patients into high-risk group (HRG) and low-risk group (LRG), according to the median RS. Then, we investigated the proportion of BLCA patients with different responses to ICI treatment between the two groups in the IMvigor210 dataset. The findings revealed that the LRG had a greater number of patients who had complete response/partial response (CR/PR) and a smaller proportion of patients who had progressing disease/stable disease (PD/SD) (**Supplementary Figure 5A**). Furthermore, those in the SD/PD group exhibit greater risk ratings contrasted with those in the CR/PR group (**Supplementary Figure 5B**). Also, KM survival analysis manifested that low RS patients have a longer survival compared to those with a high-risk score (**Supplementary Figure 5C**), which aligns with our earlier findings. Nevertheless, those belonging to the HRG had a more favorable response to Bacillus Calmette-Guérin (BCG) immunotherapy compared to those in the LRG (**Supplementary Figures 6A, B**). This may be attributed to several factors. For instance, the high-risk group may exhibit heightened immune activation and increased sensitivity to immunotherapy, whereas the low-risk group may demonstrate greater immune tolerance and more effective immune-evasion mechanisms.

### 3.12 Chemotherapeutic response analysis

**Figure 10:**
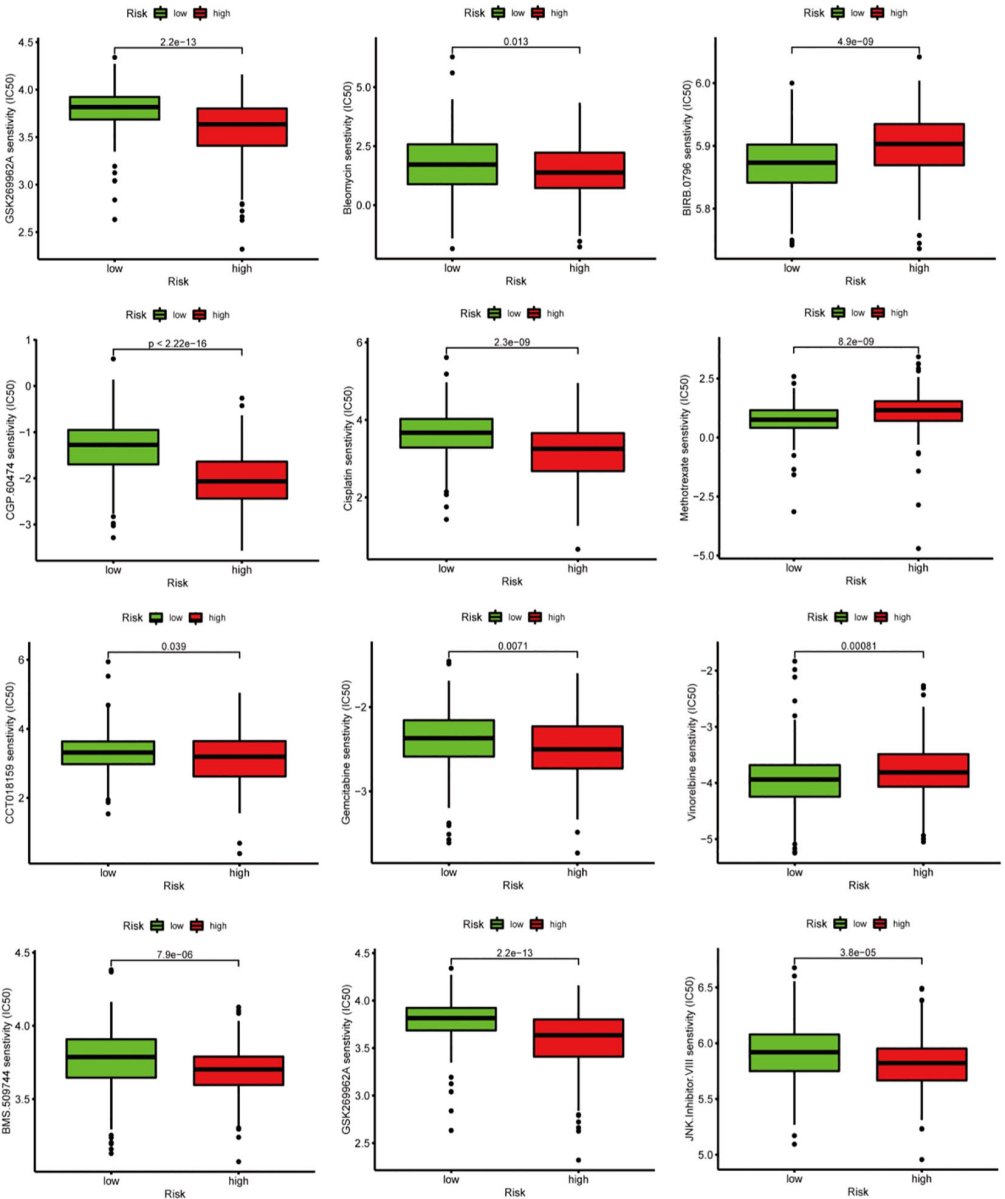
Chemotherapy sensitivity analyses between high-risk group (HRG) and low-risk group (LRG).

We conducted a further assessment of the susceptibility to chemotherapeutic treatments in two distinct groups. The analysis of the GDSC database revealed that low-risk group (LRG) had higher IC50 values for chemotherapy drugs such as GSK269962A, BMS.509744, CCT018159, CGP.60474, and JNK.Inhibitor. VIII, Cisplatin, Doxorubicin, and Gemcitabine, contrasted with high-risk group (HRG). Conversely, HRG had increased IC50 values for BIRB.0796, Methotrexate, and Vinorelbine, contrasted with LRG (**Figure 10**).

### 3.13 ATP2B4 has been shown to significantly impact the proliferation of bladder cancer cells, as demonstrated by both *in vitro* and *vivo* studies

**Figure 11:**
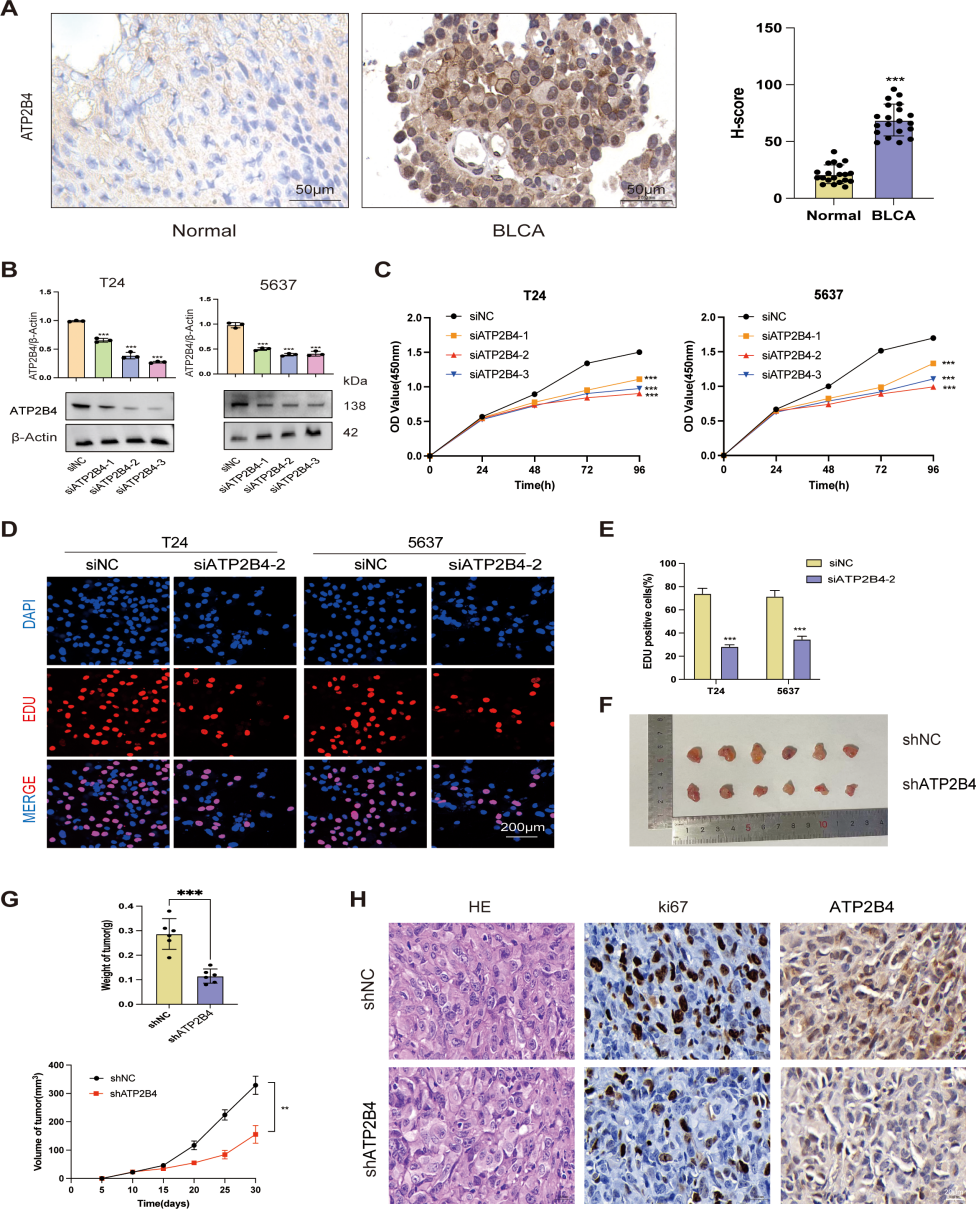
ATP2B4 is overexpressed in tumor tissues, and its suppression reduces bladder cancer growth *in vitro* and *in vivo*. **(A)** IHC representation chart showed ATP2B4 expression in normal bladder tissue and BLCA tissue. Scale bar: 50μm. **(B)** WB detection of the relative expression of ATP2B4 in control, NC and siATP2B4 groups. **(C)** Results of silencing ATP2B4 expression at different time points of CCK-8:24, 48, 72, 96h. **(D, E)** Edu assay showing proliferating cells (T24 and 5637); Edu (red) and DAPI (blue) staining. Scale bar: 200μm. **(F)** Photographs of excised T24 xenografts. **(G)** Analysis of xenograft tumor weight and volume across two groups. **(H)** HE staining of the tumor and IHC staining of Ki67, ATP2B4. Bar =20μm. *P < 0.05; **P < 0.01; ***P<0.001.

ATP2B4 functions as a germplasm membrane calcium pump, utilizing ATP hydrolysis to extrude Ca2+ from cells, thereby maintaining a low intracellular Ca^2+^ concentration (25). This protein is integral to the calcium signaling pathway, affecting cellular processes such as metabolism, proliferation, migration, and apoptosis through its regulation of intracellular Ca^2+^ levels (26). While ATP2B4 has been documented in other types of cancer (25, 27), its impact on BLCA remains unreported. In this study, we examined ATP2B4 expression in BLCA tissues obtained from local patients. We collected samples from twenty primary BLCA patients, including both cancerous and corresponding paracancerous epithelial tissues. Detailed clinical data for each patient are provided in **Supplementary Table S1**. Immunohistochemistry (IHC) analysis revealed significantly elevated ATP2B4 protein levels in the cancerous tissues compared to the paracancerous tissues (**Figure 11A**). Furthermore, the application of siRNA effectively disrupted ATP2B4 mRNA expression, as confirmed by Western blot analysis (**Figure 11B**). The CCK-8 assay indicated that cell viability was reduced following the suppression of ATP2B4 expression (**Figure 11C**). Furthermore, Edu proliferation assays revealed that the downregulation of ATP2B4 expression significantly decreased the Edu-positive rate in BLCA cells (**Figures 11D, E**). These results suggest that siRNA-mediated silencing of ATP2B4 inhibits BLCA cell proliferation. To elucidate the role of ATP2B4 in BLCA, we further investigated the effects of increased ATP2B4 levels on T24 cells in *in vivo* experiments. An orthotopic tumor mouse model of BLCA was established using T24 cells transfected with either vector or ATP2B4 knockdown lentivirus. The downregulation of ATP2B4 resulted in a significant reduction in the size and weight of T24 orthotopic tumors (**Figures 11F, G**). Immunohistochemical staining was conducted to assess the expression of Ki67 and ATP2B4 in tumor tissues (**Figure 11H**), and the results were consistent with those obtained *in vitro*.

### 3.14 The downregulation of ATP2B4 induced oxidative stress and compromised mitochondrial function in BLCA cells

**Figure 12:**
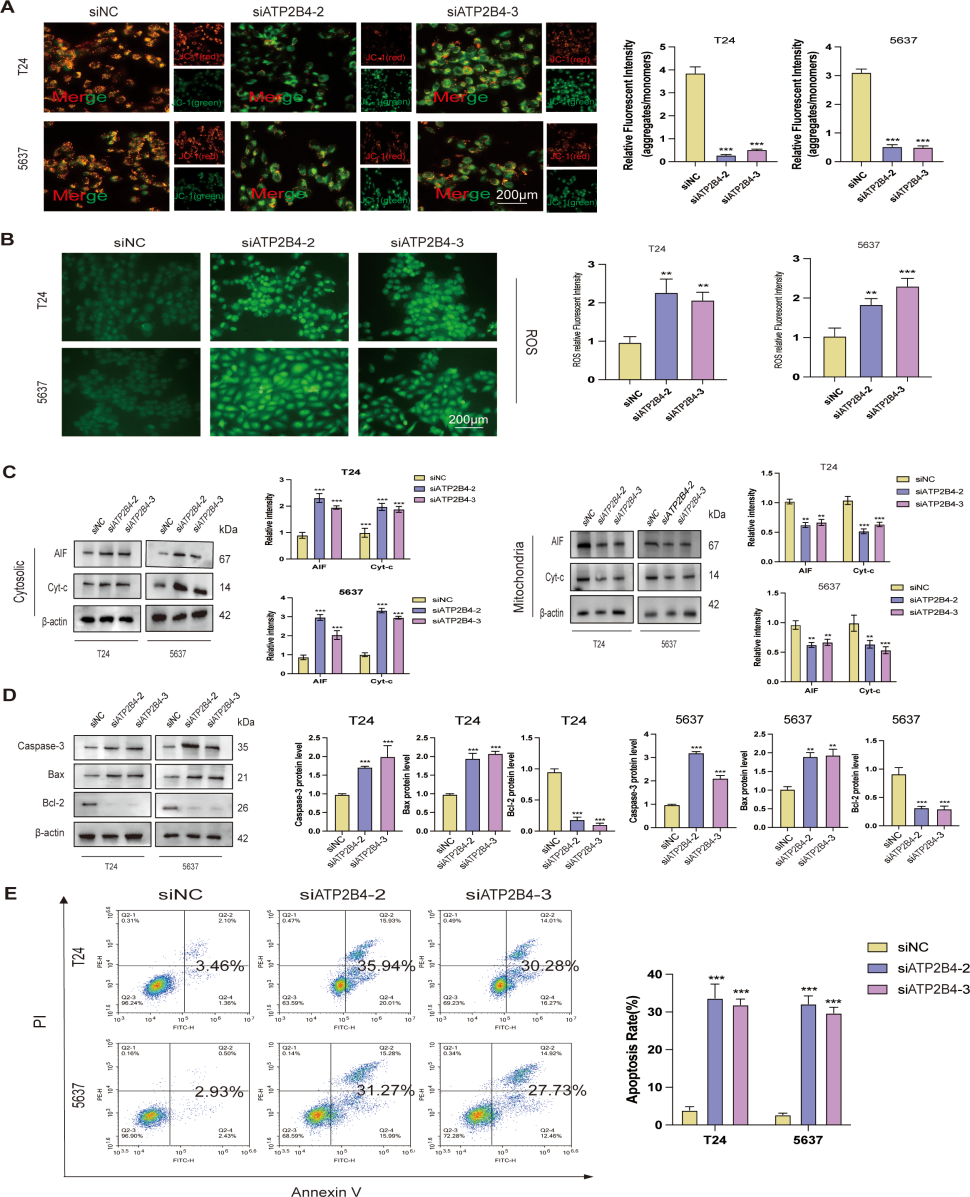
Downregulation of ATP2B4 Caused Oxidative Stress and Impaired Mitochondrial Function in BLCA cells. Fluorescence images and quantitative analysis of **(A)**JC-1 and **(B)**ROS. **(C)** Immunoblots and quantitative analysis of AIF and Cyt-c in the cytosolic and mitochondria. **(D)** Protein expression of caspase-3, Bax and Bcl-2. **(E)** Apoptosis of T24 and 5637 assessed via flow cytometry. **P < 0.01 and ***P<0.001 versus the siNC group.

To elucidate the mechanisms by which siATP2B4 induces cell death in BLCA cells, we examined the fluorescence of JC-1, reactive oxygen species (ROS), and the expression of several apoptosis-related proteins. The study demonstrated that siATP2B4 treatment resulted in a decreased red (aggregates) to green (monomers) fluorescence intensity ratio of JC-1 (**Figure 12A**), indicating a reduction in mitochondrial membrane potential (MMP) and potentially compromising mitochondrial membrane integrity. Furthermore, siATP2B4 treatment was associated with a significant increase in ROS fluorescence intensity compared to the siNC group (**Figure 12B**), suggesting an elevated production of ROS. Western blot analysis revealed a significant upregulation in the protein expression levels of cytoplasmic cytochrome c (Cyt-c) and apoptosis-inducing factor (AIF) in the siATP2B4-treated groups compared to the siNC group. Conversely, the protein expression levels of Cyt-c and AIF were significantly reduced in the mitochondria of BLCA cells treated with siATP2B4 (**Figure 12C**). The findings indicate that siATP2B4 treatment promotes the translocation of cytochrome c (Cyt-c) and apoptosis-inducing factor (AIF) from the mitochondria to the cytosol in bladder cancer (BLCA) cells. This observation is further corroborated by a significant upregulation of caspase-3 and Bax proteins, coupled with a reduction in Bcl-2 levels in the siATP2B4-treated groups (**Figure 12D**). The overall apoptotic rate, which includes both late-phase (First Quadrant) and early-phase apoptosis (Fourth Quadrant), exhibited a dose-dependent increase in response to siATP2B4 treatment (**Figure 12E**). This release is a well-established critical step in the apoptotic pathway, suggesting a potential mechanism by which siATP2B4 induces apoptosis in BLCA cells.

### 3.15 The knockdown of ATP2B4 markedly facilitates the translocation of Ca^2+^ from the cytoplasm to the mitochondria and activates the VDAC1/MCU signaling pathway

**Figure 13:**
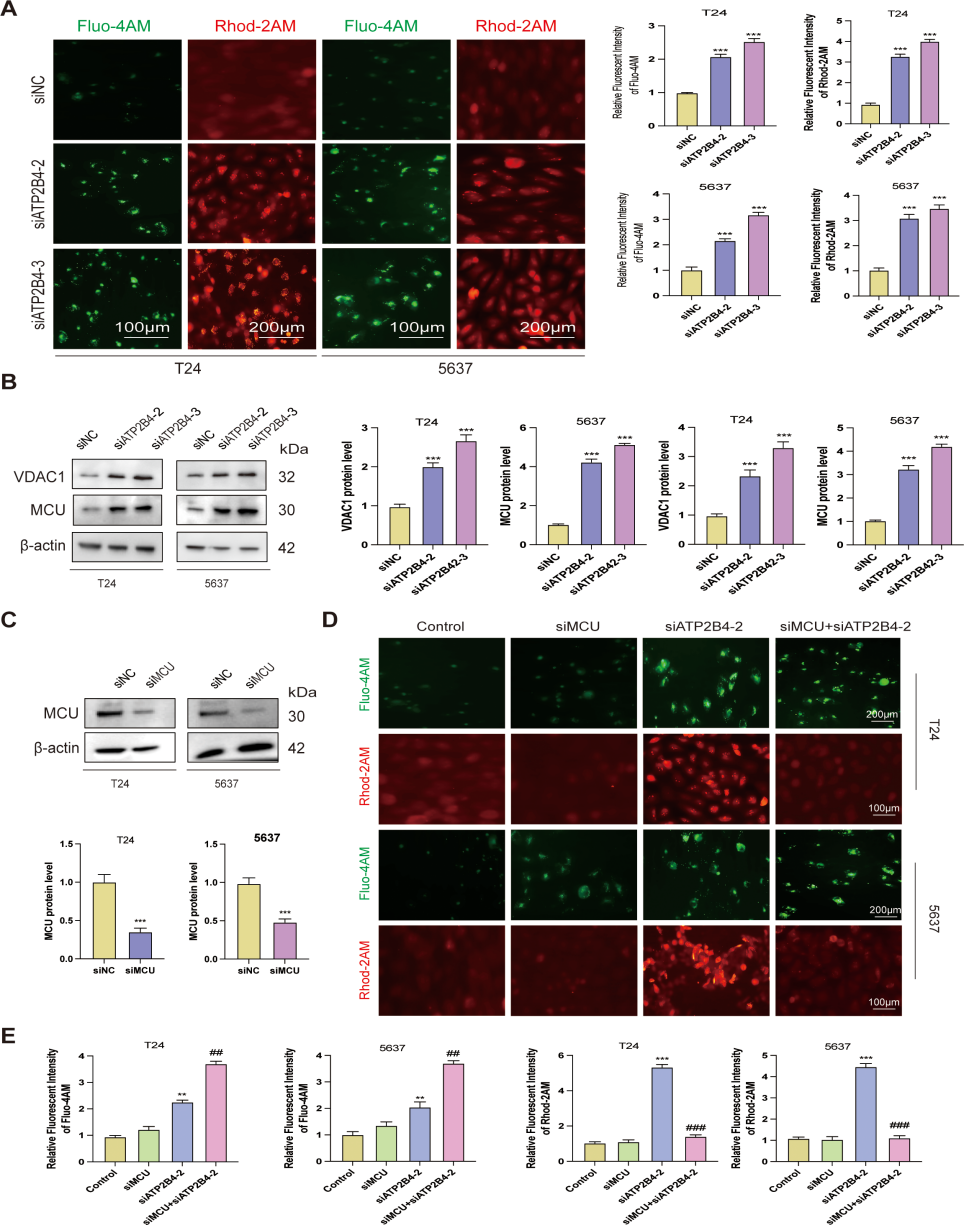
Down-regulating ATP2B4 inhibits the Ca^2+^ from the cytoplasm to mitochondria and stabilizes the mitochondrial membrane potential via the VDAC1/MCU signaling pathway. **(A)** Fluorescence images and quantitative analysis of [Ca^2+^]cyt and [Ca^2+^]mit, detected using Fluo-4 AM (Scale bar: 200μm) and Rhod-2 AM (Scale bar: 100μm), respectively. **(B)** Immunoblot analysis of VDAC1, and MCU. **(C)** T24 and 5637 cells treated with siMCU for 24 hours, immunoblot analysis of MCU. **(D, E)** T24 and 5637 cells treated with siMCU and/or siATP2B4 for 24 hours, stained with Fluo-4 AM (Scale bar: 200μm) and Rhod-2 AM (Scale bar: 100μm). **P < 0.01 and ***P<0.001 versus the control group. #*P* < 0.05 and ##*P* < 0.01 compared with the siATP2B4 group.

As a crucial intracellular second messenger, cytoplasmic calcium concentration ([Ca^2+^]i) plays a pivotal regulatory role in tumor cell migration and invasion. Utilizing the fluorescent probe Fluo-4 AM, we precisely quantified cytoplasmic calcium levels ([Ca^2+^]cyt). The experimental data indicated that T24 and 5637 cells subjected to siATP2B4 treatment exhibited a significant increase in green fluorescence intensity compared to the control group, signifying elevated cytoplasmic calcium levels. Concurrently, the assessment of mitochondrial calcium concentration ([Ca^2+^]mit) using Rhod-2 AM revealed a substantial increase in mitochondrial calcium fluorescence intensity in siATP2B4-treated cells (**Figure 13A**). These findings compellingly demonstrate that siATP2B4 treatment results in elevated cytoplasmic Ca^2+^ concentration, which in turn facilitates an increase in mitochondrial calcium levels. Voltage-dependent anion channels (VDACs), located in the outer mitochondrial membrane (OMM), function as essential conduits for intercompartmental communication. Subsequently, Ca^2+^ ions are transported into the mitochondrial matrix via the mitochondrial calcium uniporter (MCU). Western blot analysis confirmed that siATP2B4 treatment significantly upregulated the expression of both VDAC1 and MCU (**Figure 13B**). To further elucidate the specific role of ATP2B4 in modulating the VDAC1/MCU pathway, MCU expression was effectively knocked down using siMCU (**Figure 13C**). Functional assays demonstrated that siMCU efficiently inhibited Ca^2+^ transfer from the cytoplasm to the mitochondria. This was evidenced by a marked increase in the green fluorescence intensity of Fluo-4 AM and a corresponding decrease in the red fluorescence intensity of Rhod-2 AM in the siMCU + siATP2B4 co-treatment group (**Figures 13D, E**). Collectively, these findings suggest that siATP2B4 activates the VDAC1/MCU pathway by increasing cytoplasmic calcium concentration, thereby enhancing mitochondrial calcium uptake.

### 3.16 The knockdown of MCU has the potential to attenuate siATP2B4-induced mitochondrial calcium accumulation and apoptosis

**Figure 14:**
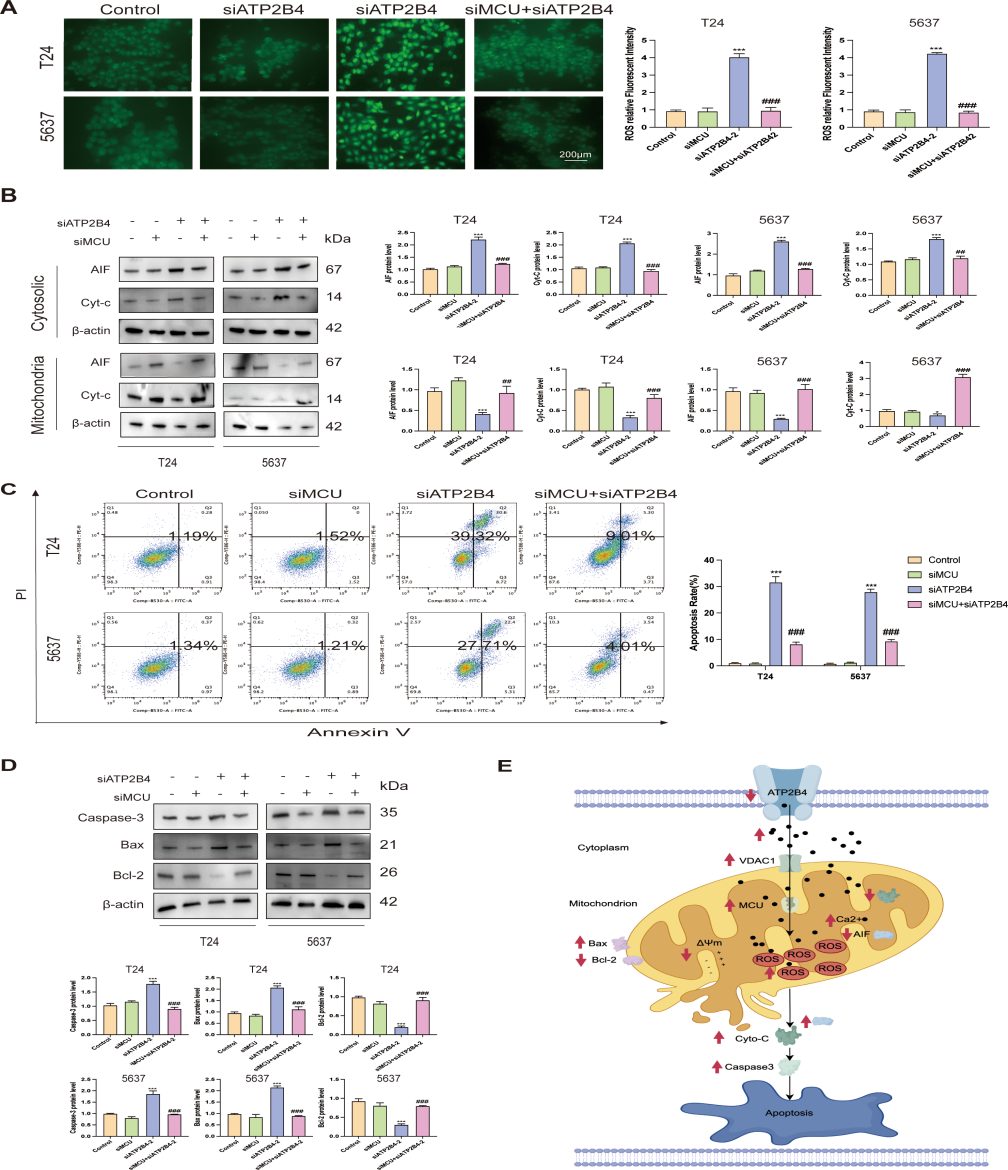
The knockdown of MCU can weaken siATP2B4-induced mitochondrial calcemia and apoptosis. **(A)** T24 and 5637 cells treated with siMCU and/or siATP2B4 for 24 hours, stained with ROS. Bar: 200 µm. **(B)** Immunoblot analysis of AIF, Cyt-c in the cytosolic and mitochondria. **(C)** Apoptosis of T24 and 5637 assessed via flow cytometry. **(D)** Immunoblot analysis of caspase-3, Bax and Bcl-2. **(E)** schematic of the working model. **P < 0.01 and ***P<0.001 versus the control group. #*P* < 0.05 and ##*P* < 0.01 compared with the siATP2B4 group.

To elucidate the function of ATP2B4 in modulating calcium (Ca^2+^) transport between the cytoplasm and mitochondria through the VADC1-MCU signaling pathway, we employed siMCU to downregulate MCU expression in bladder cancer (BLCA) cells. This approach resulted in a notable decrease in the fluorescence intensity of reactive oxygen species (ROS) (**Figure 14A**), effectively mitigating the ROS increase induced by siATP2B4. Moreover, this intervention inhibited the translocation of apoptosis-inducing factor (AIF) and cytochrome c (Cytc) from the mitochondria to the cytoplasm (**Figure 14B**), significantly reducing ATP2B4 depletion-induced apoptosis (**Figure 14C**). Additionally, siMCU treatment led to a suppression of caspase-3 and Bax expression while enhancing Bcl-2 expression (**Figure 14D**). These findings suggest that siATP2B4 may activate the VADC1-MCU calcium signaling pathway by elevating cytoplasmic Ca^2+^ concentration, thereby promoting calcium influx into the mitochondria, resulting in mitochondrial calcium overload, increased ROS production, and subsequent cellular apoptosis (**Figure 14E**). In bladder cancer cells, ATP2B4 is essential for maintaining low basal levels of cytoplasmic Ca^2+^.

## 4 Discussion

BLCA is a frequently occurring malignant tumor in the urologic tumors. It is known for its fast growth and resistance to therapy, leading to high rates of death and illness. Multiple studies have shown the pivotal significance of Ca^2+^ in carcinogenesis, metastasis, and the effectiveness of treatments for cancer. Most studies aimed to investigate the function of Ca^2+^ in cancer progression, migration, and metastasis, and few papers have systematically analyzed the expression and prognostic value of Ca^2+^-linked genes in tumors, particularly in BLCA. As far as we know, this is the first to comprehensively ascertain the Ca^2+^ metabolism pattern of BLCA and characterize their biological outcomes, particularly the Ca^2+^-mediated immune landscape, through an integrated bioinformatics approach.

This work included a thorough bioinformatics analysis to examine the expression patterns of 178 genes associated with Ca^2+^ in BLCA and their relationship with prognosis. We have detected the distinctive characteristics of six genes associated with Ca^2+^ that are linked to the advancement and results of patients with BLCA. Furthermore, we have confirmed the uniqueness of this genetic pattern in an independent dataset. Additionally, we discovered that the risk score (RS) had independent prognostic significance for BLCA. Moreover, multivariate ROC analysis has further shown that the RS’s accuracy in predicting prognosis exceeds that of standard clinical indicators. The results of GSEA demonstrated significant enrichment of cancer- and immune-related pathways in the high-risk group (HRG), while aging- and metabolism-related pathways were mostly enriched in the low-risk group (LRG). In addition, we have also discovered that the prognostic signature may function as biomarkers for predicting the therapeutic efficacy of Bacillus Calmette-Guérin (BCG) and immune checkpoint inhibitor (ICI) immunotherapy, as well as chemotherapy. These findings collectively suggest that ATP2B4 precisely regulates intracellular calcium homeostasis in bladder cancer cells via the VDAC1/MCU pathway, and alterations in its function significantly impact tumor cell survival and fate. The six calcium-linked genes (CaRGs) highlighted, including ATP2B4, BDKRB2, EDNRA, PDGFRA, EGFR, and ADCY7, have been emerged to be contributed in developing cancer. ATP2B4, also called PMCA4, was mainly related to maintaining the homeostasis of intracellular calcium by facilitating the outflow of intracellular Ca^2+^ (28). The elevated expression of ATP2B4 was correlated with cancer cell differentiation by regulating the efflux of calcium (29). Roberts et al. have reported that ATP2B4 might facilitate the cancer cells growth and migration in a calcium-dependent way (30). Many studies have confirmed that bradykinin accelerates cancer progression through various pathways by activating BDKRB2 (31). Li et al. found that the IRX1/BDKRB2 signaling pathway was involved in peritoneal spreading and lung metastasis of gastric cancer (10). Over-expression of EDNRA was correlated with inferior clinicopathologic features and unfavorable outcomes in various cancers, including BLCA (32, 33). PDGFRA was implicated in the malignant progression of tumors (34). PDDGFA, which was overexpressed in BLCA, showed a significant prognostic value in BLCA patients (35). ADCY7, a crucial member of adenylate cyclase family, was involved in inflammatory responses and immune responses by accelerating the transition of adenosine triphosphate (ATP) to cyclic adenosine monophosphate (cAMP) (36, 37). Zeng et al. have confirmed that ADCY7 was up-regulated in numerous tumors and correlated with immune microenvironment and prognosis by integrated bioinformatics analysis (38). ATP2B4 has been selected as the focal point of this study due to its pivotal role as a calcium pump located on the cell membrane, where it is instrumental in the regulation of Ca^2+^ homeostasis and serves as a crucial regulatory factor within the calcium signaling pathway. Dysfunction of ATP2B4 has been implicated in a range of diseases, including cancer, neurodegenerative disorders, and cardiovascular conditions, thereby underscoring its importance as a primary gene of interest for investigating the Ca^2+^ signaling pathway. In contrast, the genes BDKRB2 and EDNRA, while involved in the activation of phospholipase C (PLC) through G protein-coupled signaling pathways leading to increased intracellular Ca^2+^ concentrations, have a comparatively limited role in the calcium signaling pathway. Similarly, PDGFRA, EGFR, and ADCY7 are recognized for their significant contributions to cell proliferation, differentiation, and survival, yet their direct influence on the calcium signaling pathway is relatively minor. Consequently, ATP2B4 is prioritized for research to facilitate a more comprehensive understanding of the mechanisms underlying Ca^2+^ signaling.

Numerous investigations have proven the essential function of Ca^2+^ in tumor microenvironment and significant influence of immune cell infiltrating on prognosis (39–43). To further examine the disparity in infiltrating immune cells between two groups, we employed TIMER, CIBERSORT-ABS, XCELL, EPIC, QUANTISEQ, MCP-counter, and CIBERSORT algorithms to accurately measure the quantities of immune cells. The results indicated that antineoplastic immune cells, including CD4^+^ T cells, follicular helper T (Tfh) cells, and plasma cells, were increased in low-risk group (LRG), while pro-cancer immune cells encompassing M0 macrophages, M2 macrophages, Tregs, neutrophils, and cancer-associated fibroblasts (CAFs) were heightened in high-risk group (HRG). Tfh cells, a specific subset of CD4+ T cells, were characterized by germinal center response and have been reported to be linked to favorable outcomes in multiple cancers (44, 45). Moreover, some investigations have confirmed that the existence of Tfh cells was also correlated with tertiary lymphoid structures (TLS), which were widely believed to be associated with longer survival (46). Cytokine interleukin 21 (IL-21) secreted by Tfh cells could accelerate the maturation of B cells into plasma cells that enhance the antineoplastic immune response by producing tumor antigen-specific antibodies (47, 48). The accumulation of Tfh cells facilitated the antitumor immunity of CD8+ T cells and improved the therapeutic effect of anti-PD-L1 treatment (45). Meanwhile, the antineoplastic effect of Tfh cells can be inhibited by PD-L1/PD-1 signaling (49). Increasing evidence has demonstrated that CAFs were the predominant cellular constituents of the TME in tumors and expedited the malignant progression of cancers (50, 51). Exosomes derived from CAFs facilitated the metastasis and attenuated the chemosensitivity of BLCA cells by PTEN-mediated Wnt/β-catenin signaling pathways (52). FAP-positive CAFs might promote immunosuppression and resistance to immune checkpoint inhibitor (ICI) immunotherapy (53). To the best of our knowledge, the Tregs-mediated immunosuppressive microenvironment was characterized via secreting immunosuppressive cytokines (IL-10, -35, TGF-β) or expressing immune checkpoints (PD-1, LAG-3, CTLA-4, PD-L1, and TIGIT) (54, 55). M2 macrophages were remodeled by cytokines (IL-10, IL-4, and IL-13) and thus expedited immune escape (56, 57). All these were consistent with our results that the levels of cytokines (IL-10, TGF-β, and IL-13) and immunological checkpoints (PD-1, LAG-3, CTLA-4, PD-L1, HAVCR2, and TIGIT) were shown to be higher in the HRG. The adverse prognosis experienced by patients within the HRG may be ascribed to the immunosuppressive microenvironment.

To get a deeper understanding of the molecular pathways linked to the prognostic signature, we conducted a GSEA comparing two groups. The research discovered that the high-risk group (HRG) emerged a notable increase in cancer-correlated pathways, while the low-risk group (LRG) had a substantial increase in metabolism-linked pathways. Prior research has manifested that the TGF-β signaling pathway is mostly active in immune-excluded or immunological-inflamed phenotypes and remodeled the TME to facilitate immune evasion by restraining T cell infiltration (22), which was in accordance with our result that TGF‐β signaling pathway was particularly activated in HRG. Taken together, our results inaugurated a novel approach to improve the therapeutic effect of immune checkpoint inhibitor (ICI) immunotherapy.

Although it was widely believed that immune checkpoint inhibitor (ICI) based immunotherapies had scored tremendous advancement in clinical application for various types of cancer, including BLCA, only a fringe of patients showed a response to ICI treatment (58, 59). Therefore, identification of biomarkers for predicting ICI response was of great clinical significance. Several biomarkers, including the expression of PD-1/PD-L1, tumor mutation burden (TMB), and tumor-infiltrating lymphocytes, have been identified as the predictive factors for the selection of patients who can maximize the benefit of ICI therapy (60–63). Previous studies also indicated that microbiome and sex hormone levels can improve the efficacy of ICI treatment (64, 65). Furthermore, Jiang et al. have shown that the genetic pattern known as Tumor Immune Dysfunction and Exclusion (TIDE) may be employed as a reliable indication for forecasting the effectiveness of ICI treatment (66). In our recent research, we discovered that our distinctive marker was linked to the response to ICI treatment. We observed that the low-risk group (LRG) patients manifested a more favorable prognosis and response. These findings showed that a calcium-based signature can be employed to forecast the response and prognosis of ICI treatment in BLCA patients. Conversely, our research revealed that those classified as high-risk group (HRG) showed a greater level of responsiveness to Bacillus Calmette-Guérin (BCG) immunotherapy. The difference in response to ICI immunotherapy and BCG immunotherapy among the two groups might be correlated with TMB. In our investigation, TMB was obviously negatively linked to risk score (RS). The higher TMB represented a better response to the ICI therapy in BLCA (61–67). This phenomenon can be attributed to multiple factors. For example, individuals within the high-risk group may display elevated immune activation and heightened sensitivity to immunotherapy. In contrast, those in the low-risk group may exhibit enhanced immune tolerance and more efficient immune-evasion mechanisms. Nevertheless, the lower TMB represented the preferred response to the BCG therapy (68). Furthermore, individuals classified as HRG had heightened sensitivity to commonly used chemotherapeutic medicines, including bleomycin, cisplatin, and gemcitabine. Conversely, LRG exhibited more sensitivity to vinorelbine and methotrexate. We also found that several small molecule compounds, such as tomatine, tyloxapol, Prestwick-1084, mepenzolate bromide, calcium pantothenate, nisoxetine, carbarsone, isoconazole, hydrocotarnine, and harmine, could increase the therapeutic implications of BLCA patients. Based on existing research and data, among these ten compounds, some have been identified as regulators of calcium channels, while others lack direct evidence and may represent speculative connections. Tomatine, an alkaloid extracted from tomatoes, has been shown to stimulate AMPK phosphorylation through CaMKKβ activation in response to increased intracellular Ca^2+^ concentrations (69). Nisoxetine, a selective serotonin reuptake inhibitor used in the treatment of depression, primarily targets the serotonin system; however, studies suggest it may indirectly influence calcium signaling by modulating neuronal excitability. Harmine, a natural alkaloid with diverse pharmacological effects, has been demonstrated to regulate cellular physiological functions by affecting intracellular Ca^2+^ levels (70). Currently, there is no direct evidence supporting the ability of other small molecule compounds to regulate calcium channels.

This study constitutes the inaugural demonstration that ATP2B4 modulates intracellular Ca^2+^ concentration in bladder cancer cells through the VDAC1/MCU signaling pathway. The degree of malignancy in cell line 5637 is relatively high, whereas cell line T24 exhibits a comparatively lower degree of malignancy. This distinction facilitates a more comprehensive examination of the differential responses of cells with varying malignancy levels to the Ca^2+^ signaling pathway, particularly when assessing its impact on bladder cancer cell behavior. The study identified variations in the activation and transmission of the Ca^2+^ signaling pathway between T24 and 5637 cells (71–75). These differences provide a foundation for investigating the specific role of the Ca^2+^ signaling pathway in distinct bladder cancer cell lines and contribute to a deeper exploration of its precise mechanisms in the pathogenesis and progression of bladder cancer. Consequently, T24 and 5637 were selected as the subjects for this study. The maintenance of intracellular calcium homeostasis is crucial for normal cellular physiological functions; however, it is often disrupted in cancer cells, consequently affecting tumorigenesis, progression, angiogenesis, and metastasis (13, 14). The plasma membrane calcium ATPase (PMCA/ATP2B) family is responsible for maintaining low cytosolic calcium concentrations by actively transporting Ca^2+^ ions from the cytoplasm to the extracellular environment. Our observations indicate that ATP2B4 is significantly overexpressed in bladder cancer tissues, and its downregulation induces apoptosis in bladder cancer cells. The voltage-dependent anion channel 1 (VDAC1), predominantly situated in the outer mitochondrial membrane, is integral to the regulation of mitochondrial homeostasis and is involved in various cell death pathways, including apoptosis, autophagy, and ferroptosis (18, 19). The mitochondrial calcium uniporter (MCU) complex mediates the influx of Ca^2+^ from the cytoplasm into the mitochondria (20), a process regulated by cytosolic calcium concentration. Through siRNA-mediated knockdown of ATP2B4, we observed a significant elevation in cytosolic calcium levels, which activated the VDAC1/MCU signaling axis and enhanced mitochondrial calcium uptake. This led to mitochondrial calcium overload, a reduction in mitochondrial membrane potential, increased production of reactive oxygen species (ROS), and ultimately induced apoptosis in bladder urothelial carcinoma (BLCA) cells. Moreover, knockdown of MCU effectively inhibited calcium translocation into the mitochondria and alleviated the ROS accumulation and apoptotic effects induced by ATP2B4 depletion. These findings collectively suggest that ATP2B4 precisely regulates intracellular calcium homeostasis in bladder cancer cells via the VDAC1/MCU pathway, and alterations in its function significantly impact tumor cell survival and fate.

While our research has achieved notable advancements, certain limitations remain. First, despite implementing stringent screening criteria and correcting for batch effects, the possibility of selection bias cannot be entirely ruled out. Second, the nomogram integrates risk scores with age but omits other clinical parameters, which may potentially diminish its predictive accuracy, support for clinical decision-making, generalization performance, and the interpretability of the classification tree. Third, further functional characterization of ATP2B4 is essential, particularly through *in vivo* models, to enhance our understanding of its role in the tumor microenvironment. Fourth, Current studies mainly focus on the association between TMB and calcium signaling genes, but have not yet delved into the direct impact of specific gene mutations on this association. Lastly, additional preclinical and clinical validations are necessary for the ten potential therapeutic compounds identified via CMAP analysis.

## 5 Conclusions

Ultimately, we have created and validated a novel signature employing six genes that are linked to Ca^2+^ signaling. This signature was shown to be independently connected with the BLCA patient prognosis. Furthermore, the prognostic signature demonstrated exceptional efficacy in forecasting the response to chemotherapy and immunotherapy in BLCA patients, which might serve as an effective biomarker for providing individualized treatment of BLCA cancer patients. The multifaceted role of ATP2B4 as a pivotal tumor-promoting gene in BLCA encompasses the regulation of tumor cell proliferation and apoptosis, as well as the participate of calcium ion transport within tumors. Targeting ATP2B4 and its associated pathways may offer novel therapeutic approaches for the treatment of BLCA patients.

## Data Availability

The original contributions presented in the study are included in the article/**Supplementary Material**. Further inquiries can be directed to the corresponding author.
