# Supplementary material for: Prognostic significance of calcium signaling-related genes in bladder cancer and the role of ATP2B4 in regulating mitochondrial calcium ion levels via the VDAC1/MCU pathway
Source: Front Immunol. 2026 Feb 6;17:1561666. doi: 10.3389/fimmu.2026.1561666 (PMC12920588; doi:10.3389/fimmu.2026.1561666)
Supplement: Supplementary file 1 [file DataSheet1.pdf]

Supplementary Material

Supplementary Figures

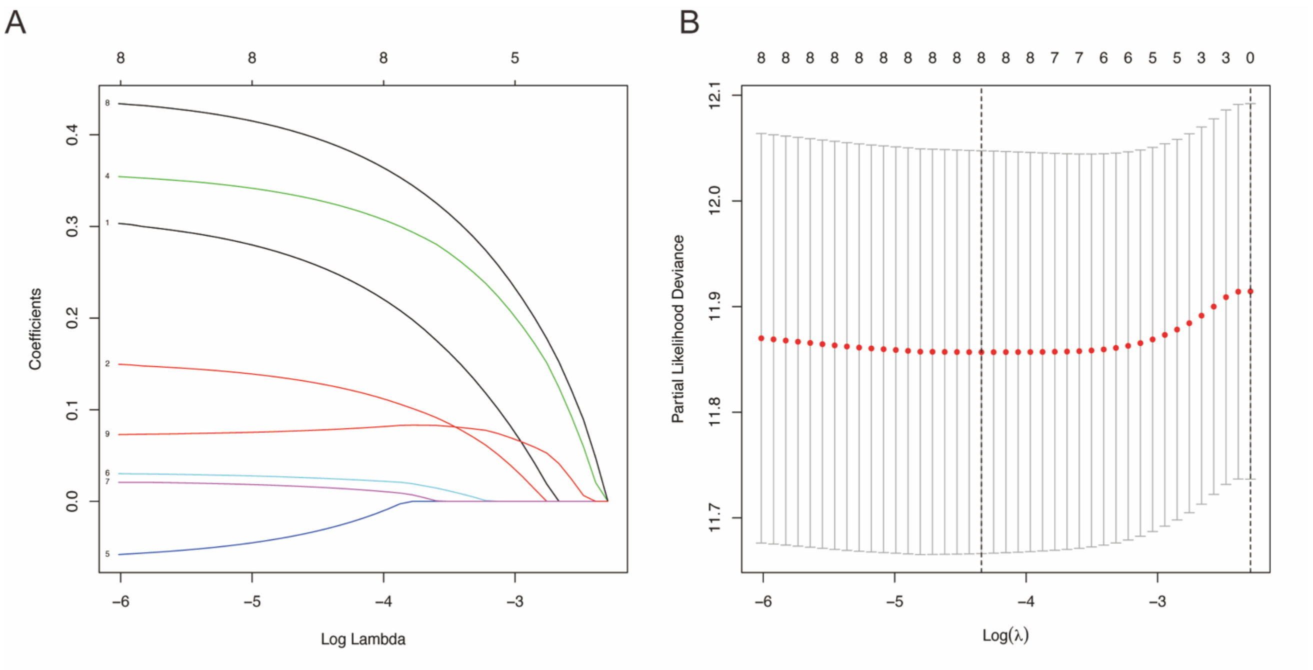

Supplementary Figure 1. (A) LASSO regression coefficient plot. (B)10-fold cross-validation plot for LASSO regression dimensionality reduction.

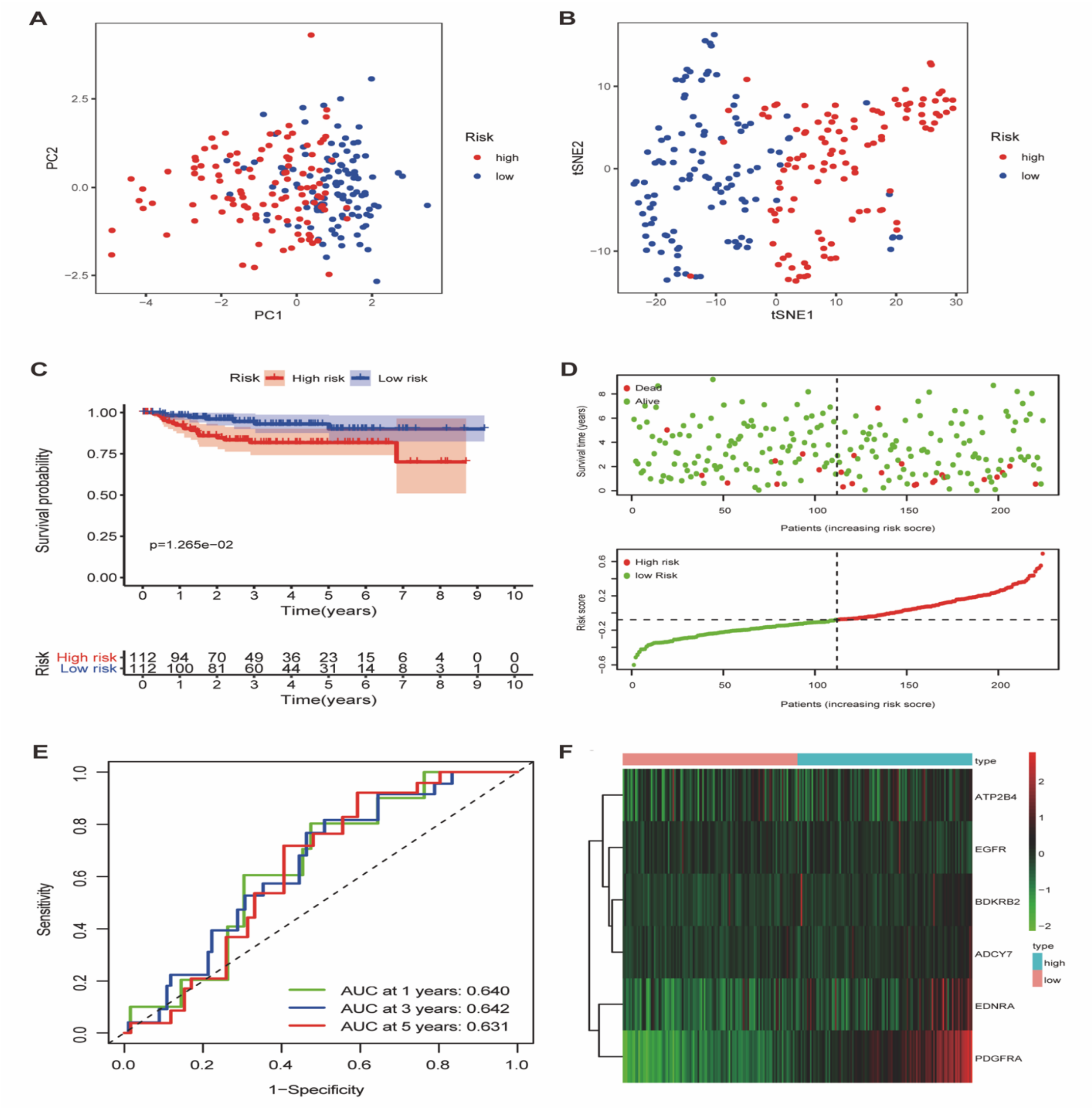

Supplementary Figure 2. Construction and validation of the prognostic signature in GSE32894 dataset (A) PCA analyses; (B) t-SNE analyses; (C)Kaplan-Meier survival analysis of BLCA patients between high-risk groups and low-risk groups; (D)Distribution of risk score and different patterns of survival status and survival time between the high- and low- risk groups; (E)Time-independent receiver operating characteristic (ROC) analysis of the risk score predicting the overall survival; (F) Heatmap displayed the distribution of six genes between the high- and low-risk groups.

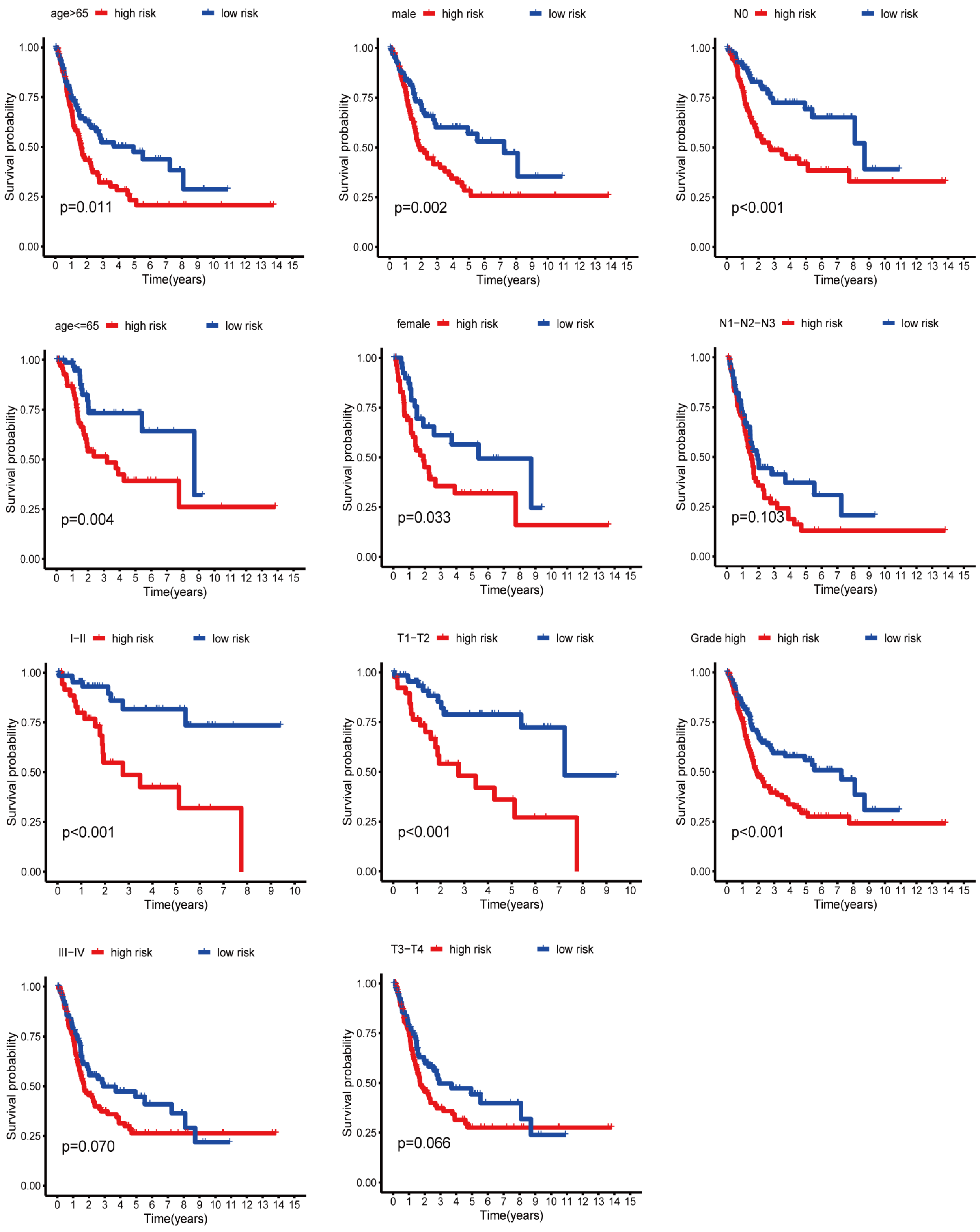

Supplementary Figure 3 Subgroup analyses stratified by various clinical features (T stage, N stage, grade, TNM stage, age, and gender)

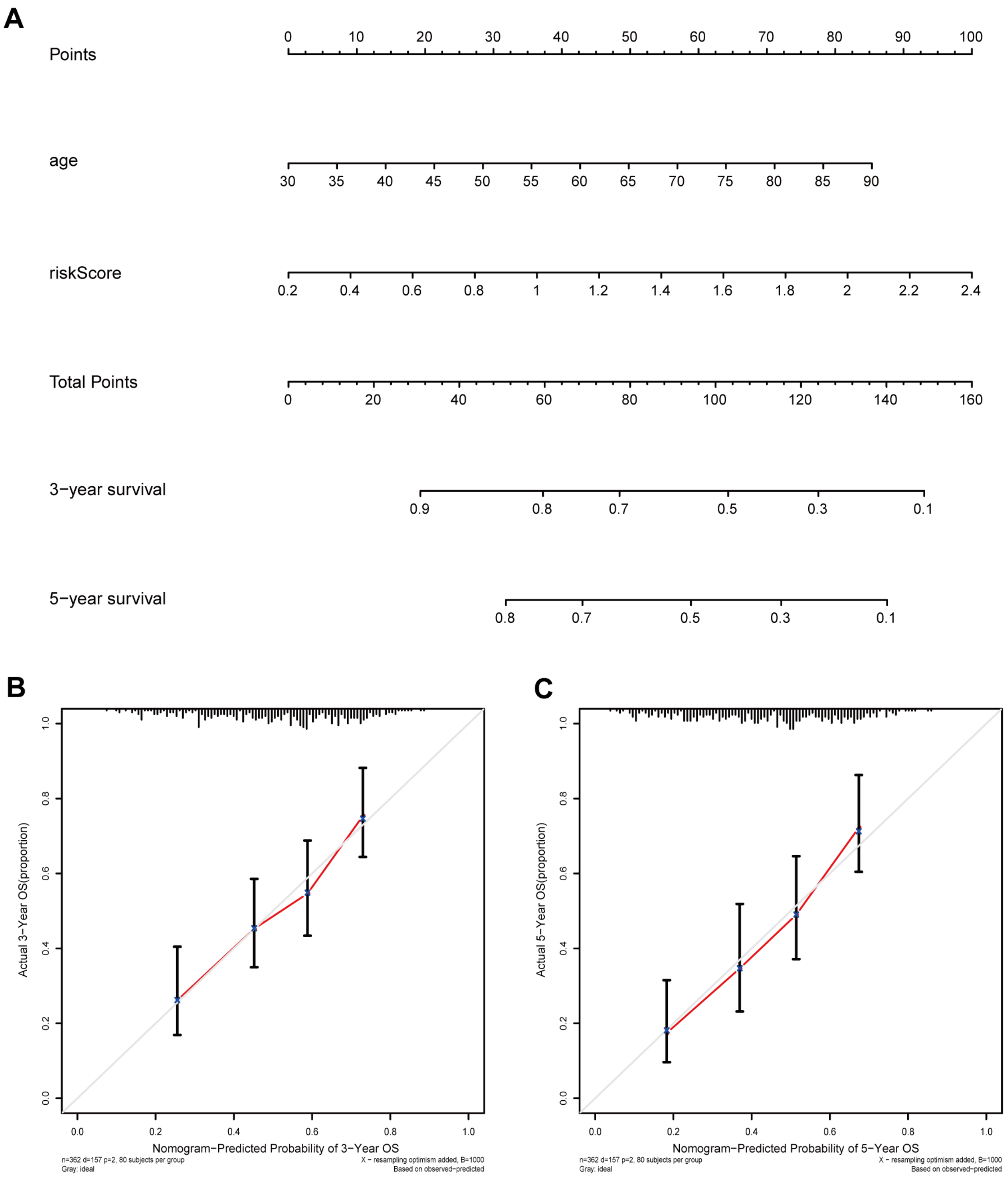

Supplementary Figure 4 (A)Nomogram for anticipating 3- or 5-year OS; (B-C) The calibration plots for anticipating 3-and 5-year OS.

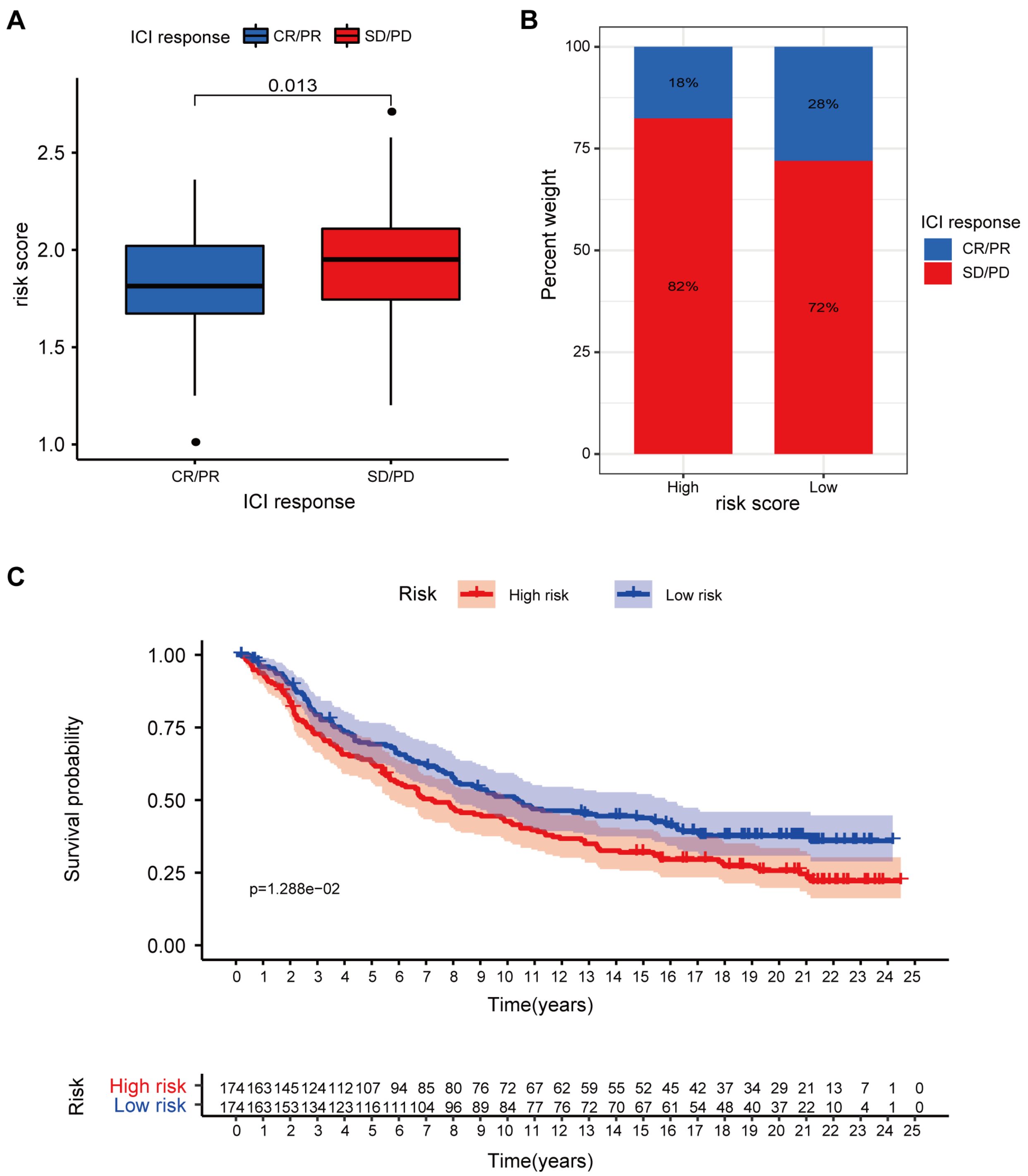

Supplementary Figure 5. (A and B) Comparison between the risk score and ICI therapy response groups showed that a lower risk score might predict better response. (C) The Kaplan-Meier estimates of overall survival for patients categorized as HRG and LRG manifested that the LRG had superior survival outcomes.

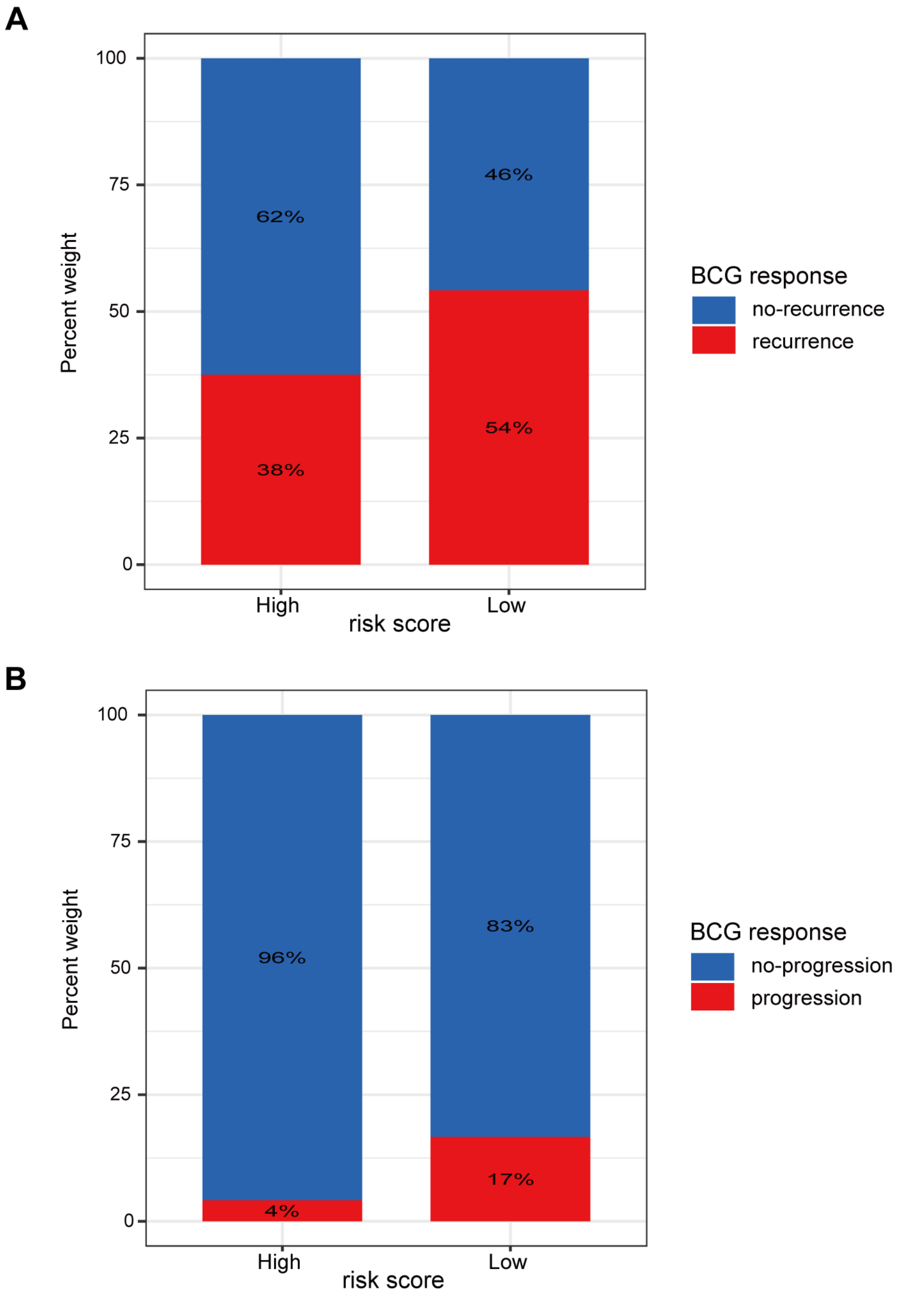

Supplementary Figure 6. (A)Comparison between the RS and BCG therapy response manifested that a greater RS might predict lower recurrence; (B)Comparison between the RS and BCG therapy response emerged that a greater RS might predict lower progression.

Supplementary Table S1 Detailed clinical data for each patient.

Clinical data														
**ID**	**Age**	**Disease**	**Sex**	**T**	**N**	**M**	**Stage**	**Grade**	**newTumor**	**Smoking**	**Radiation**	**Neoadjuvant**	**Therapy**	**OS**
**Patient 1**	79	BLCA	MALE	T3	N0	M0	III	G2	NA	No	No	No	NA	0
**Patient 2**	62	BLCA	FEMALE	T3	N1	M0	Ⅳ	G2	NA	No	No	No	NA	0
**Patient 3**	72	BLCA	MALE	T2	N1	M0	Ⅳ	G2	NA	No	No	No	NA	0
**Patient 4**	72	BLCA	MALE	T1	N0	M0	I	G1	NA	No	No	No	NA	0
**Patient 5**	70	BLCA	MALE	T4	N0	M0	III	G3	NA	No	No	No	NA	0
**Patient 6**	68	BLCA	MALE	T2	N0	M0	II	G2	NA	No	No	No	NA	0
**Patient 7**	65	BLCA	FEMALE	T3	N0	M0	III	G2	NA	No	No	No	NA	0
**Patient 8**	67	BLCA	MALE	T3	N1	M0	Ⅳ	G3	NA	No	No	No	NA	0
**Patient 9**	62	BLCA	MALE	T1	N0	M0	I	G2	NA	No	No	No	NA	0
**Patient 10**	59	BLCA	FEMALE	T2	N0	M0	II	G2	NA	No	No	No	NA	0
**Patient 11**	76	BLCA	MALE	T3	N0	M0	III	G3	NA	No	No	No	NA	0
**Patient 12**	74	BLCA	FEMALE	T3	N1	M0	Ⅳ	G3	NA	No	No	No	NA	0
**Patient 13**	58	BLCA	MALE	T4	N0	M0	III	G3	NA	No	No	No	NA	0
**Patient 14**	79	BLCA	MALE	T2	N0	M0	II	G2	NA	No	No	No	NA	0
**Patient 15**	69	BLCA	FEMALE	T3	N0	M0	III	G2	NA	No	No	No	NA	0
**Patient 16**	67	BLCA	MALE	T2	N0	M0	II	G2	NA	No	No	No	NA	0
**Patient 17**	66	BLCA	MALE	T3	N0	M0	III	G3	NA	No	No	No	NA	0
**Patient 18**	72	BLCA	MALE	T2	N0	M0	II	G2	NA	No	No	No	NA	0
**Patient 19**	81	BLCA	MALE	T2	N0	M0	II	G2	NA	No	No	No	NA	0
**Patient 20**	78	BLCA	FEMALE	T4	N1	M0	Ⅳ	G3	NA	No	No	No	NA	0

Supplementary Table S2 siRNA and shRNA target sequences.

Gene	Sequences (5'- -3')
siATP2B4-1	GAUGCACUGACCCAGAUUAAUTT
siATP2B4-2	GGGCAUCCAUUACCGUCAAAUTT
siATP2B4-3	CCGGACUAUCUGCAUAGCUUATT
shATP2B4	GATGCACTGACCCAGATTAAT
siMCU	AUCAGGCAUUGUGGAAUAUAATT
