# Supplementary figures and images for: Prognostic significance of calcium signaling-related genes in bladder cancer and the role of ATP2B4 in regulating mitochondrial calcium ion levels via the VDAC1/MCU pathway
Source: Front Immunol. 2026 Feb 6;17:1561666. doi: 10.3389/fimmu.2026.1561666 (PMC12920588; doi:10.3389/fimmu.2026.1561666)

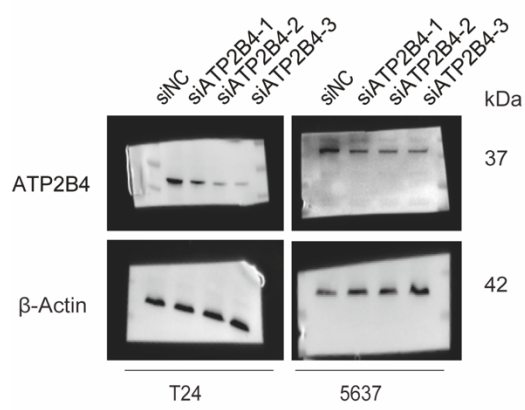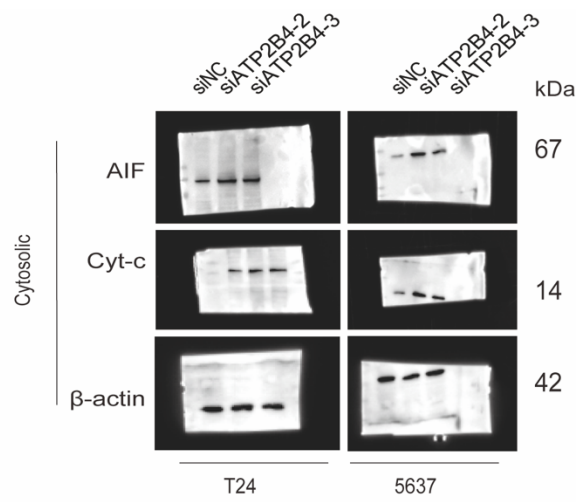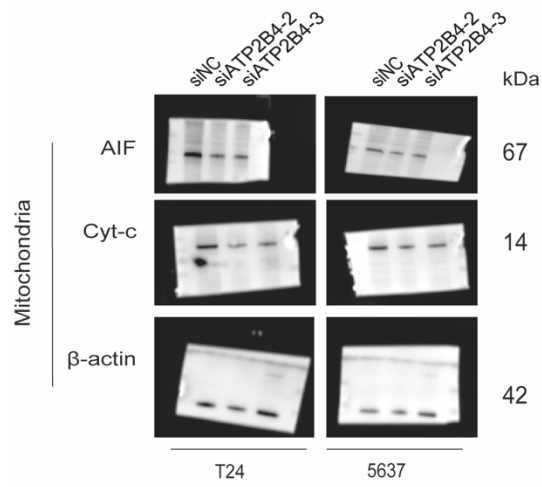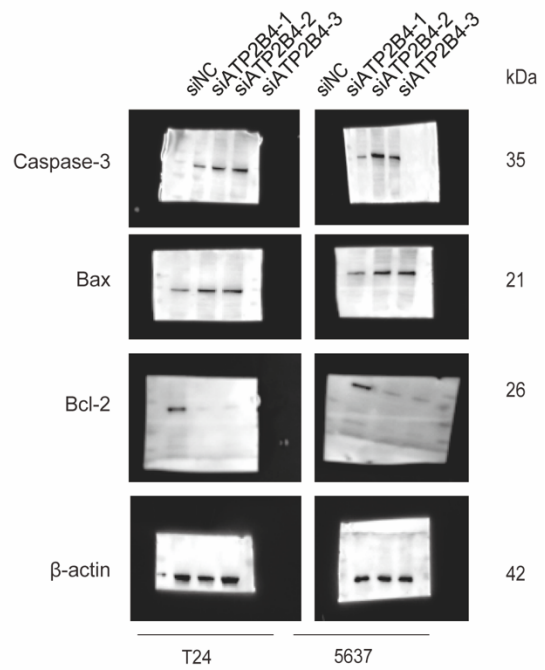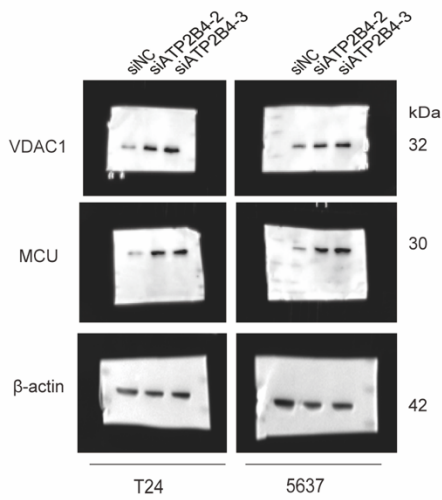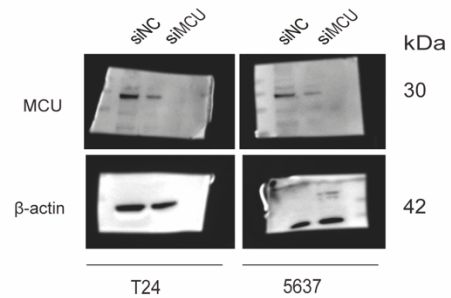

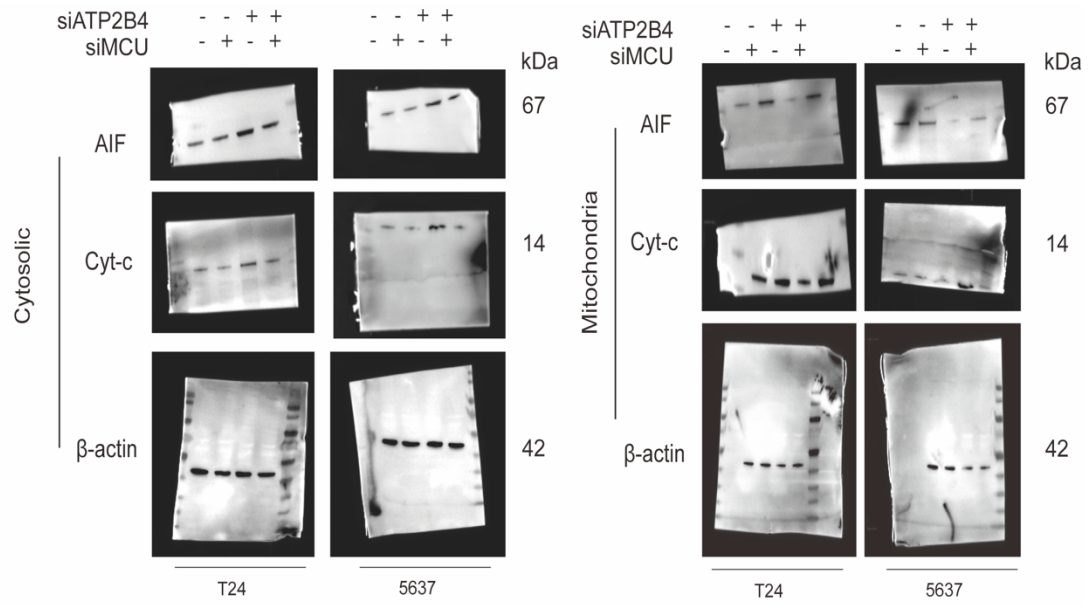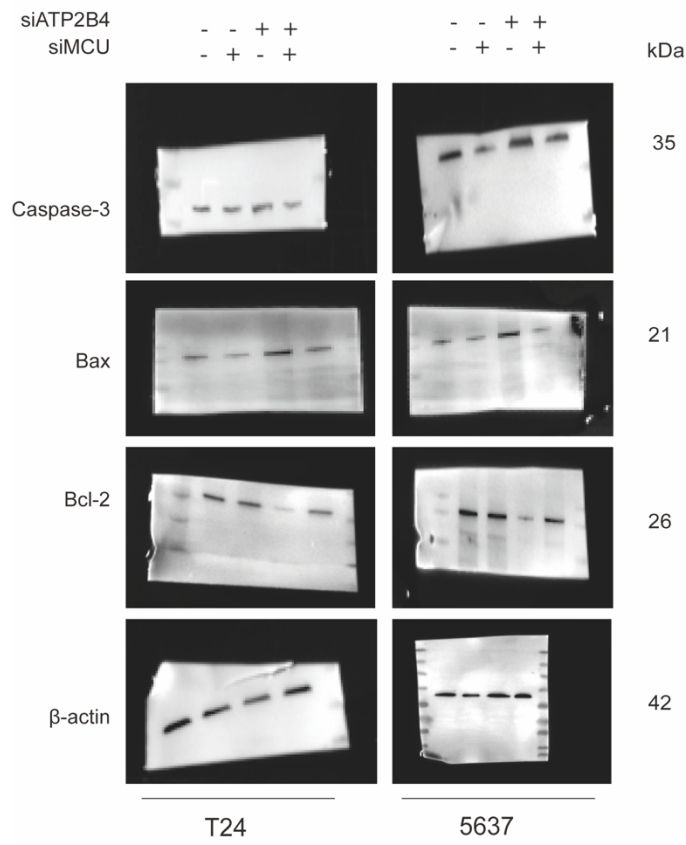

Supplement: Supplementary file 2 [file DataSheet2.pdf]
